# Hyperarid soil microbial community response to simulated rainfall

**DOI:** 10.3389/fmicb.2023.1202266

**Published:** 2023-09-14

**Authors:** Cecilia Demergasso, Julia W. Neilson, Cinthya Tebes-Cayo, Roberto Véliz, Diego Ayma, Daniel Laubitz, Albert Barberán, Guillermo Chong-Díaz, Raina M. Maier

**Affiliations:** ^1^Biotechnology Center “Profesor Alberto Ruíz”, Universidad Católica del Norte, Antofagasta, Chile; ^2^Department of Environmental Science, University of Arizona, Tucson, AZ, United States; ^3^Department of Geology, Faculty of Engineering and Geological Sciences, Universidad Católica del Norte, Antofagasta, Chile; ^4^Department of Mathematics, Faculty of Sciences, Universidad Católica del Norte, Antofagasta, Chile; ^5^Steele Steele Children’s Research Center, Department of Pediatrics, University of Arizona, Tucson, AZ, United States

**Keywords:** hyperarid, soil microbiome, soil wetting, extremophiles, oligotrophic microbes, Atacama Desert, mixotroph

## Abstract

The exceptionally long and protracted aridity in the Atacama Desert (AD), Chile, provides an extreme, terrestrial ecosystem that is ideal for studying microbial community dynamics under hyperarid conditions. Our aim was to characterize the temporal response of hyperarid soil AD microbial communities to *ex situ* simulated rainfall (5% g water/g dry soil for 4 weeks) without nutrient amendment. We conducted replicated microcosm experiments with surface soils from two previously well-characterized AD hyperarid locations near Yungay at 1242 and 1609 masl (YUN1242 and YUN1609) with distinct microbial community compositions and average soil relative humidity levels of 21 and 17%, respectively. The bacterial and archaeal response to soil wetting was evaluated by 16S rRNA gene qPCR, and amplicon sequencing. Initial YUN1242 bacterial and archaeal 16S rRNA gene copy numbers were significantly higher than for YUN1609. Over the next 4 weeks, qPCR results showed significant increases in viable bacterial abundance, whereas archaeal abundance decreased. Both communities were dominated by 10 prokaryotic phyla (Actinobacteriota, Proteobacteria, Chloroflexota, Gemmatimonadota, Firmicutes, Bacteroidota, Planctomycetota, Nitrospirota, Cyanobacteriota, and Crenarchaeota) but there were significant site differences in the relative abundances of Gemmatimonadota and Chloroflexota, and specific actinobacterial orders. The response to simulated rainfall was distinct for the two communities. The actinobacterial taxa in the YUN1242 community showed rapid changes while the same taxa in the YUN1609 community remained relatively stable until day 30. Analysis of inferred function of the YUN1242 microbiome response implied an increase in the relative abundance of known spore-forming taxa with the capacity for mixotrophy at the expense of more oligotrophic taxa, whereas the YUN1609 community retained a stable profile of oligotrophic, facultative chemolithoautotrophic and mixotrophic taxa. These results indicate that bacterial communities in extreme hyperarid soils have the capacity for growth in response to simulated rainfall; however, historic variations in long-term hyperaridity exposure produce communities with distinct putative metabolic capacities.

## Introduction

The Atacama Desert (AD) provides an ideal laboratory for studying microbial habitability under hyperarid conditions and identifying associated unique survival strategies. The AD soils reach ages exceeding 10 million years and have undergone an exceptionally long and protracted period of aridity (Hartley and Chong, 2002) that is rare on the rest of the planet. In addition, the occurrence and distribution of salts have allowed the characterization of the AD as a unique Saline Domain (Chong-Diaz et al., 2020). This extreme environment has attracted diverse research interests. For example, bio-signatures can offer insights into past or present life on surfaces such as early Mars, and guide astrobiology research on extraterrestrial surfaces (Cabrol et al., 2007; Hock et al., 2007; Certini et al., 2009; Certini and Ugolini, 2013; Warren-Rhodes et al., 2019). Related to this is interest in the strategies used by microbes to remain metabolically active over long time scales under hyperarid conditions, a topic that remains poorly understood (Davila et al., 2013; Leung et al., 2020).

Deserts are not only characterized by low precipitation levels, but precipitation events are erratic. Soil microorganisms in extreme, hyperarid deserts such as the AD and the Namib Desert in Namibia must survive protracted periods of aridity between isolated rainfall events. Data from the Namib Desert projected that surface soil microbial communities have active growth for just 184–363 h per year (Bosch et al., 2022). Similarly, soil relative humidity (RH) data recorded in the AD from 2015 to 2018 (sensors at 20 cm depth recording every 2 h) revealed average RH values of just 17–29% across eight hyperarid sites with no values recorded above 52% (Neilson et al., 2017). Areas in the AD that experience rainfall have soil RH values of 100%. Thus, AD soil microbial communities in hyperarid regions survive years to decades with no precipitation events (Neilson et al., 2017; Schulze-Makuch et al., 2018; Supplementary Figure S1 AD rainfall history). Temporal studies in deserts are rare, but it is clear that isolated rainfall events are a primary trigger controlling microbial activity (Huxman et al., 2004;Makhalanyane et al., 2015).

These extreme conditions support increasing interest in understanding the strategies for survival of the AD microbiome during prolonged periods of desiccation. Initial culture-independent techniques concluded that the hyperarid core of the AD was an environment completely devoid of life (Navarro-Gonzalez et al., 2003). But soon thereafter recoverable DNA and low cell numbers of bacteria from soils in the extreme arid core were reported showing differences between the composition of the microbial community in vegetated and unvegetated areas (Maier et al., 2004; Drees et al., 2006; Connon et al., 2007).

Later studies employing high throughput sequencing techniques facilitated the identification of associations between environmental factors, soil microbial community composition, and potential metabolic strategies under arid and hyperarid conditions. Two research studies evaluated associations between aridity gradients and microbial community composition along extensive transects. Moisture and soil electrical conductivity significantly correlated with microbial community diversity along a North–South (24–26° Latitude S) moisture gradient transect (Crits-Christoph et al., 2013). In a second study along a west–east elevational transect, moisture and temperature explained significant reductions in the diversity, evenness, and connectivity of AD soil microbial communities (Neilson et al., 2017). In the second study, the abundance of “key bacterial taxa” typically associated with the microbiome of fertile soils as well as Archaea decreased with decreasing moisture and increasing temperature. On the contrary, more abundant microbial phylotypes were phylogenetically associated with chemolithoautotrophs that obtain energy by oxidation of nitrite (NO_2_^−^), carbon monoxide (CO), iron (Fe) or sulfur (S) suggesting genetic potential for non-phototrophic primary production and geochemical cycling in these hyperarid ecosystems (Neilson et al., 2012, 2017). Strong evidence has also been found for contributions by hydrogen-oxidizing bacteria in four distinct desert soils (Australian, Namib, Gobi, and Mohave) whose activity is stimulated by hydration (Jordaan et al., 2020). Interestingly, the microbial composition of AD hyperarid soils was site-specific suggesting that specific environmental factors shape these communities.

A study of microbial activity in the AD using oxygen (O) and nitrogen (N) isotopes indicates that at least 20% of the nitrate in the driest region of the AD has been biologically cycled (Amundson et al., 2007). However, the low rate of biological N cycling, combined with very low organic matter (OM) cycling rates based on ^14^C determination, reflected exceedingly small rates of microbial processing of carbon (C) and N. An assessment of biomolecular proxies for cell adaptation and growth suggests that for the driest areas of the AD this basal metabolic activity is used for cellular repair and maintenance and there is minimal or no *in situ* microbial growth (Wilhelm et al., 2018).

Some studies have amended AD surface soils (1–10 cm) with moisture and C substrates to evaluate the heterotrophic metabolic potential of AD soil microbial communities. Emissions of carbon dioxide (CO_2_), N_2_O, and methane (CH_4_) after amendment revealed the retention of some biogeochemical-cycling capacity despite long-term deprivation (Hall et al., 2012). In addition, an exceptional rainfall event in 2017 allowed for an *in situ* analysis of hyperarid AD soil response to moisture and provided evidence of metabolically active microbial communities (Schulze-Makuch et al., 2018). In that study, biomolecules indicative of potentially active cells [e.g., presence of ATP, phospholipid fatty acids (PLFAs), metabolites, and enzymatic activity], as well as *in situ* replication rates of metagenome-assembled genomes (MAGs) were measured, despite extremely low microbial biomass and diversity. The researchers inferred that the microbial populations underwent selection and adaptation in response to their specific soil microenvironment and to the degree of aridity.

The objective of this study was to determine the capacity of AD soil microbial communities from two distinct hyperarid locations to respond to a simulated rainfall event under controlled laboratory conditions in the absence of any nutrient amendment. Specifically, the study evaluated the response of hyperarid soil microbiomes to a soil-wetting event in terms of (1) the growth capacity of bacterial and archaeal communities and (2) the dynamic changes in relative abundances of specific taxa. Functional contributions of key taxa from each community were evaluated to characterize the metabolic response of each community. Hyperarid sites YUN1242 and YUN1609 were selected for analysis based on previous environmental and microbial community characterization that showed them to have distinct microbial communities (Neilson et al., 2017). The model experiment was conducted over 30 days to quantitatively assess the temporal response of these two microbial communities to water addition using qPCR and amplicon sequencing. The qPCR analysis quantifies the metabolically active fractions of the bacterial and archaeal communities in the respective soils, whereas changes in the relative abundance of microbial populations characterized by amplicon sequencing identify the specific taxa with the capacity to respond to a rainfall event. We hypothesize that hyperarid soil microbial communities from different geographic locations in the AD will have the same response to a simulated rainfall event. Results from this study will inform our understanding of the composition, physiology and activity of resilient microorganisms inhabiting arid soils, and will help guide the search for life in terrestrial and extraterrestrial sites.

## Results

### Soil characterization

**Figure 1:**
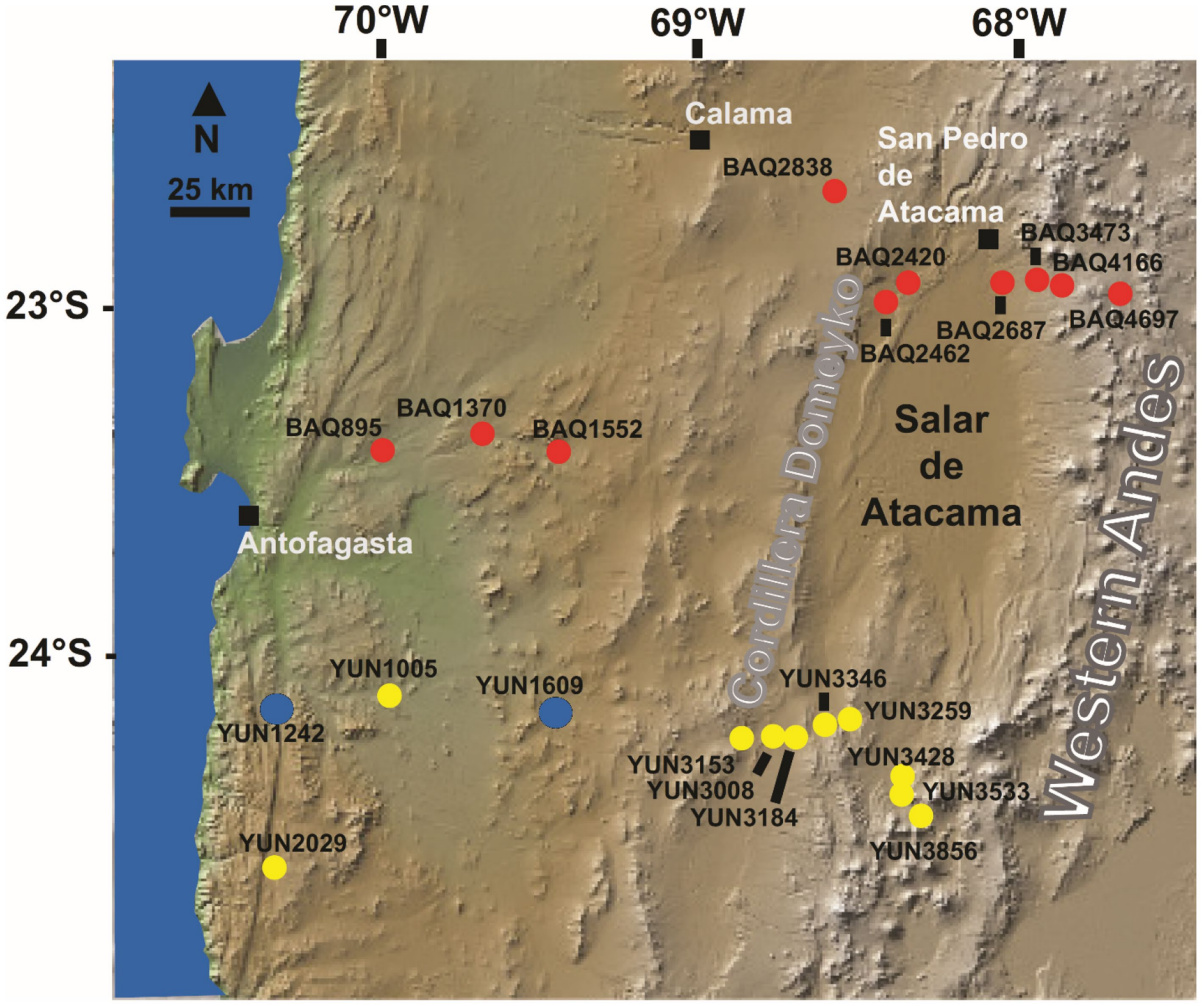
Map of the studied area indicating the locations of sampling sites YUN1242 and YUN1609 in blue at the Atacama Desert, Chile. The sites in yellow and red belong to two transects studied before (Neilson et al., 2017).

The selected sites (Figure 1), labeled YUN1242, and YUN1609, were previously classified as long-term hyperarid soil based on nitrate and sulfate profiles (Neilson et al., 2017). Nitrate and sulfate levels over 15 and 340 mmol g^−1^ soil, respectively, in the soil samples revealed the long-term hyperaridity in the Atacama Desert (Supplementary Figure S2). Higher nitrate and sulfate levels in the YUN1609 soil profile compared with YUN1242 suggest greater long-term aridity at this site. Soil organic carbon (OC) ranged from 0.02 to 0.04% (Neilson et al., 2017).

The soils in both sites were alkaline (pH 8.9 in YUN1242, and pH 8.4 in YUN1609) and were characterized by an electrical conductivity of 311.5 and 589.3 μS·cm^−1^ (Table 1) and a soil moisture of 1.71% in YUN1242, and 1.09% in YUN1609. After the rain event in 2017, the soil gravimetric water content in each sample increased to 4.196 and 6.159%, respectively (Table 1). Our records show that gravimetric moisture contents remained at these levels for at least 12 days after the 2017 rain event (Table 1). Further, elevated RH levels of 40–50% were recorded at 10–20 cm depth for more than 90 days in an alluvial fan near YUN1242 (Pfeiffer et al., 2021).

**Table 1:** Physicochemical parameters of the soil samples from January and June 2017, before and after the rain (6–7 June).

Date	Parameters	Unit	YUN1242	YUN1609
Before rain (1/25/2017)	Coordinates	Latitude (S)	24.141	24.144
		Longitude (W)	70.312	69.442
	Soil Temp.	°C	31.4	30.2
	Air RH /Temp.	%/°C	30.5/31.5	9.4/30.2
	pH		8.9	8.4
	ORP	mV	226.1	230.7
	EC	μS/cm	311.5	589.3
	UV A	μW/m^2^	6503	3019
	UV B	μW/m^2^	658.3	360.5
	UV C	μW/m^2^	14	19.75
	PAR	μmol/m^2^s	1948	2584
	Soil moisture	%	1.7380	1.0909
After rain (6/19/2017)	Soil moisture	%	4.196	6.159

RH, Relative humidity; ORP, Oxidation–reduction potential; EC, electrical conductivity; UV, ultraviolet radiation; PAR, photosynthetic active radiation.

### Bacterial and archaeal total abundances during the incubation time

**Figure 2:**
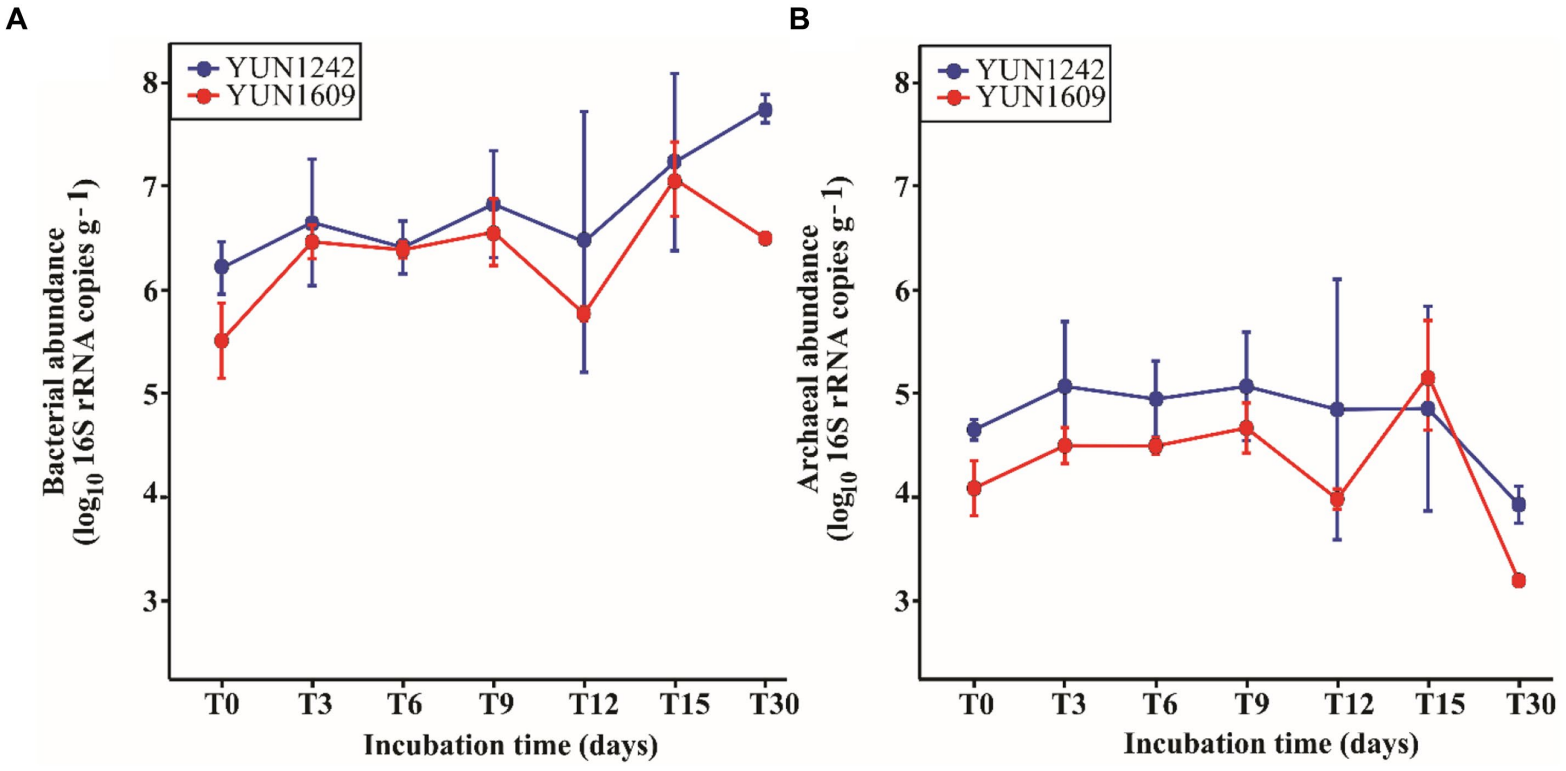
Profile of bacterial **(A)** and archaeal **(B)** 16S rRNA copies·g^−1^ (on a log 10 scale) in sampling sites YUN1242 and YUN1609 during the incubation at 5% soil moisture, 20°C, 12-h-light, and oxic environment. The means and standard deviations for observations per incubation time are represented via points and vertical lines, respectively. The raw data is in Supplementary Table S7.

The average DNA concentrations retrieved from the experiments (Supplementary Figure S3) with YUN1242 and YUN1609 samples were significantly (*p* -value < 0.001) higher than those obtained from the blanks (Supplementary Figure S4). The initial (T0) 16S rRNA copy number per gram of soil was 3.09 × 10^5^ and 1.78 × 10^6^ for Bacteria, and 1.18 × 10^4^ and 4.55 × 10^4^ for Archaea in YUN1609 and YUN1242, respectively (Figure 2). Thus, bacterial and archaeal copy numbers were significantly higher for YUN1242 soils than YUN1609 at T0 (*t*-test value of ps, 0.018 and 0.023, respectively).

The bacterial abundance response to soil wetting for both YUN1242 and YUN1609 was significantly different from that of the archaea. For bacteria, pairwise multiple comparisons using FDR correction indicated a significant increase in abundance from T0 to T30 in both sampling sites (value of *p* < 0.05). This increase was relatively steady for YUN1242. In contrast, for YUN1609 bacterial abundance increased significantly from T0 to T6 (value of *p* = 0.010), remained stable until T9, followed by a significant decrease until T12 (value of *p* = 0.014) and finally another significant increase from T12 to T30 (value of *p* < 0.001) (Figure 2 and Supplementary Table S1). Both sampling site and the incubation time were statistically significant factors (two-way repeated measures ANOVA using the log 10 bacterial total abundance as the response variable, value of *p* < 0.005).

A similar analysis of archaeal abundance indicated a significant decrease in archaeal abundance from T6 to T30 in YUN1242 (value of *p* = 0.002) and from T0 to T30 in YUN1609 (value of *p* = 0.007). Again, for YUN1242 the decrease was relatively gradual whereas for YUN1609 the copy number remained stable from T0 to T9, then decreased significantly at T12 (value of *p* = 0.019) followed by a significant rebound in abundance at T15 (value of *p* = 0.041), and then a sharp decrease again until T30 (value of *p* = 0.005) (Figure 2 and Supplementary Table S2).

### Overall community diversity and taxonomic composition

A total of 410–45,284 16S rRNA gene sequences per sample were retrieved from the extracted DNA during the wetting experiments (for T3–T30 subsamples), 690–4,617 sequences per sample from the T0 samples, and 555–1,599 sequences in both blank and reagent control experiments (Supplementary Table S3).

**Figure 3:**
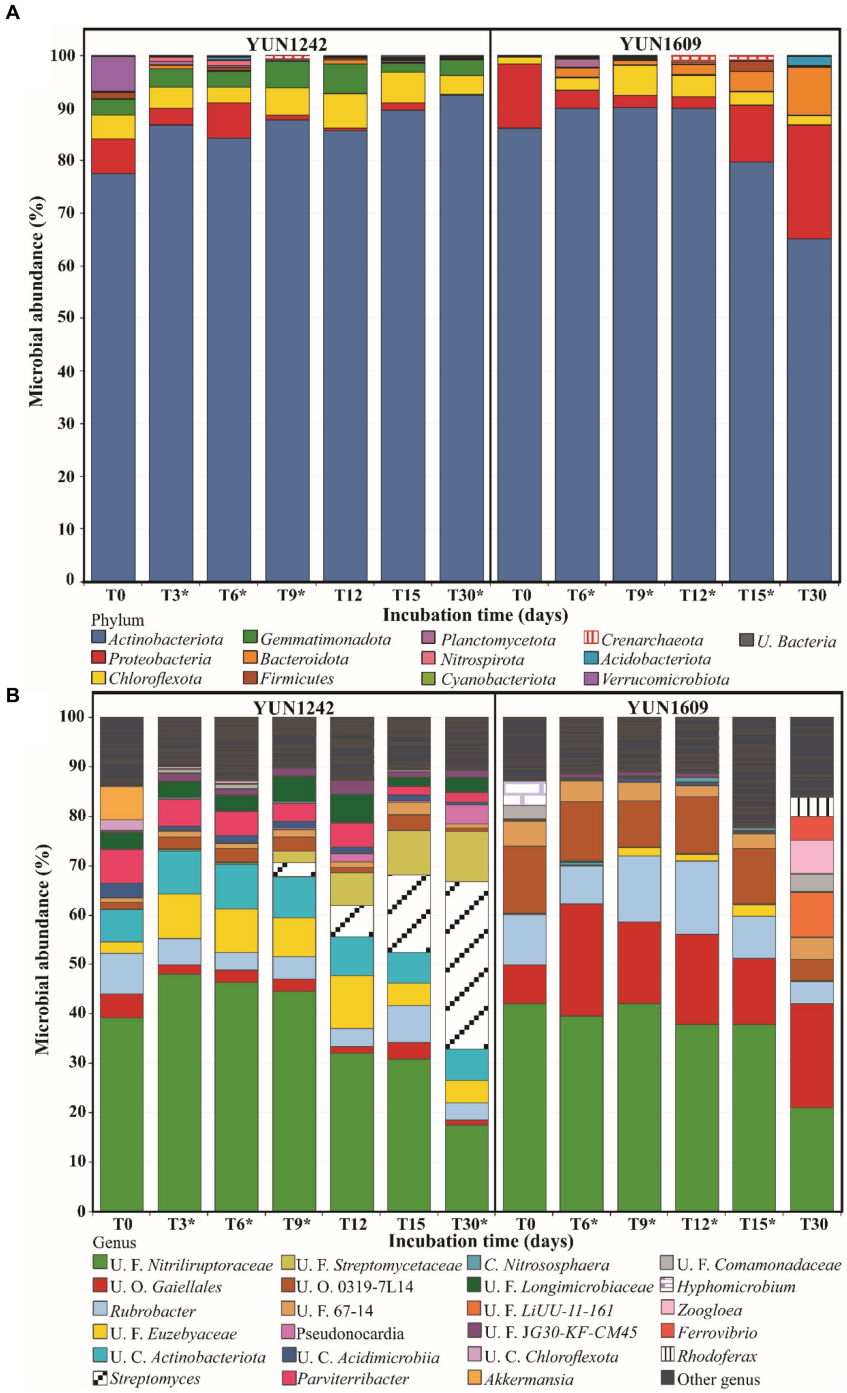
Average of relative microbial abundance at Phylum **(A)** and Genus **(B)** level versus the incubation time using YUN1242 and YUN1609 soils. * Indicates the microbial abundance’s mean of replicates. Other genus = genus that have less than 1% of abundance in all the samples.

The quality control and rarefaction (retain samples with ≥1,500 reads per sample) process provided 353,319 input sequences which resulted in 31,500 bacterial and archaeal Amplicon Sequence Variants [ASVs]. The ASVs were classified into 12 prokaryotic phyla accounting for 91% of the total sequences in the communities (Figure 3). All ASVs were classified at the kingdom level; 40.7% were assigned to genus and less than 2.3% to species.

The diversity of the YUN1242 microbial community was higher than the YUN1609 community, Shannon index averages over incubation time of 2.928 and 2.685 for YUN1242 and YUN1609, respectively (value of *p* < 0.05) (Supplementary Table S4A and Supplementary Figure S5A). The richness index showed a linear increase with time (value of *p* < 0.05) during the wetting experiments for the YUN1242 community, whereas the increase in richness for YUN1609 was not significant (value of *p* = 0.0532). Multiple linear regression analysis showed no significant difference in terms of their slopes (value of *p* > 0.05) (Supplementary Figure S6).

Eleven bacterial phyla (Actinobacteriota, Proteobacteria, Chloroflexota, Gemmatimonadota, Firmicutes, Bacteroidota, Planctomycetota, Nitrospirota, Cyanobacteriota, Acidobacteriota, Verrucomicrobiota) and one archaeal phylum (Crenarchaeota) jointly comprised 99% of the total community of both soils (samples and subsamples) (Figure 3A). The taxonomic analysis of the sequencing (control tubes) and DNA extraction (blank tubes) reagents revealed the presence of common contaminants observed in molecular techniques like *Streptococcus* (Salter et al., 2014). Although direct PCR of those tubes did not generate sequences that passed the quality score, nested PCR detected 14 contaminant ASVs that represented less than 1% of abundance on average (from 2 to 113 ASV counts) of target samples (Supplementary Information Box 1). The nested PCR approach was employed for more stringent contaminant detection as described in Supplementary Information Box 1. These contaminant sequences were eliminated in the original samples or from the re-wetting experiments.

Actinobacteriota dominated bacterial abundance ranging from 65 to 90% in the samples and subsamples from both sites (Figure 3A). Site had an important influence on Actinobacteriota distribution at the genus level. For example, the unclassified family *Nitriliruptoraceae* was predominant in the microbial communities from both sites (Figure 3B). *Rubrobacter* distribution was also similar in both sites. However, several groups were more abundant in one site or the other, e.g., the unclassified order *Gaiellales* was more abundant in YUN1609 than in YUN1242 whereas the *Streptomycetaceae* family was more abundant in YUN1242.

Chloroflexi was most represented by *Thermomicrobiales*, *Thermobaculales*, and unclassified Class Gitt-GS-136 sequences (Botero et al., 2004; Gupta et al., 2013). The *Gemmatimonadota* phylum was represented mainly by *Longimicrobia* (Figure 3B; Pascual et al., 2016) in YUN1242 and YUN1609 samples. The Proteobacteria (now Pseudomonadota) phylum was mainly represented by the order *Burkholderiales* and *Ferrovibrionales* in YUN1609, and *Rhizobiales* in YUN1242.

Noteworthy is that some bacterial phyla that are common in other dryland soils, such as Cyanobacteriota, Bacteroidota, Firmicutes, and Acidobacteriota (Leung et al., 2020) were under-represented in the samples, each of them limited to one predominant ASV.

**Figure 5:**
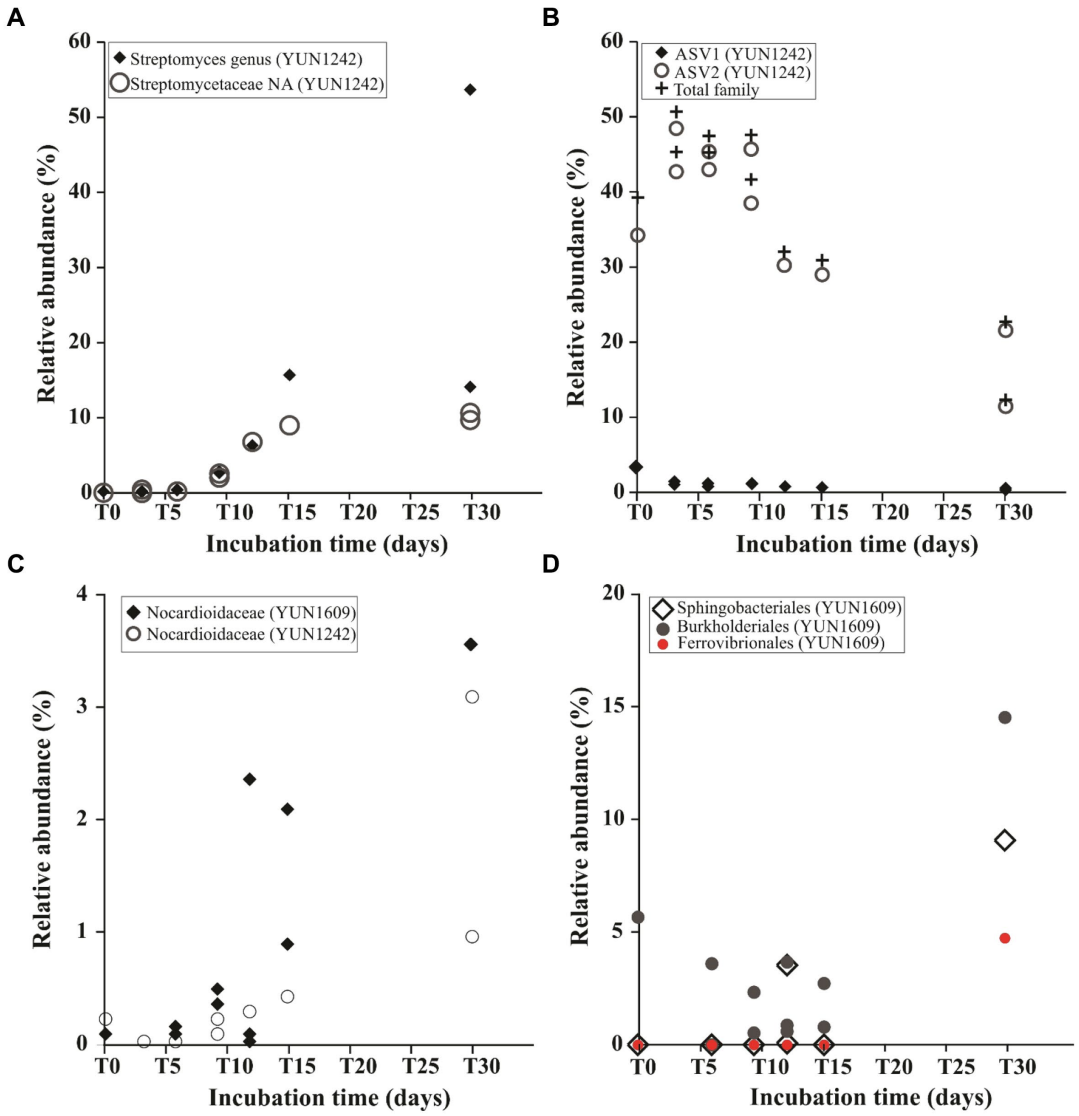
Relative abundance dynamics of taxa during the wetting experiments from the phylum *Actinobacteriota* **(A–C)**: *Streptomyces* genus and non-assigned (NA) *Streptomycetaceae* family in YUN1242 **(A)**; Predominant ASVs from *Nitriliruptoraceae* family in YUN1242 **(B)**; *Nocardioidaceae* in YUN1609 and YUN1242 samples **(C)**. Relative abundance dynamics of *Sphingobacteriales*, *Burkholderiales* and *Ferrovibrionales* in YUN1609 **(D)**. Note that each point represents the relative abundance in a single replicated flask.

An ASV occurrence diagram revealed that only 1.4% of the identified ASVs belonged to the core community (present at T0 and all subsequent time points for both YUN1242 and YUN1609). This core microbial community group was comprised primarily of *Rubrobacter*, *Thermobaculum*, unclassified *Nitriliruptoraceae*, *Gaiellales*, *Nocardioidaceae*, *Longimicrobiaceae*, and *Frankiales* (Supplementary Figure S7C and Supplementary Table S5). The change caused by wetting is further evidenced by the fact that 84 and 87.6% of the ASVs present from T3 to T30 in YUN1242, and T6 to T30 in YUN1609, respectively, were not detected in the corresponding T0 YUN1242 and YUN1609 samples (Supplementary Figures S7A,B). A particular group of interest are ASVs belonging to the genus *Streptomyces* in the YUN1242 community that were not detected in T0 samples but became dominant by T30 (Figures 3B 4B 5A). Similarly, in YUN1609, the *Sphingobacteriales* order appeared at T30 (Figure 5D).

**Figure 4:**
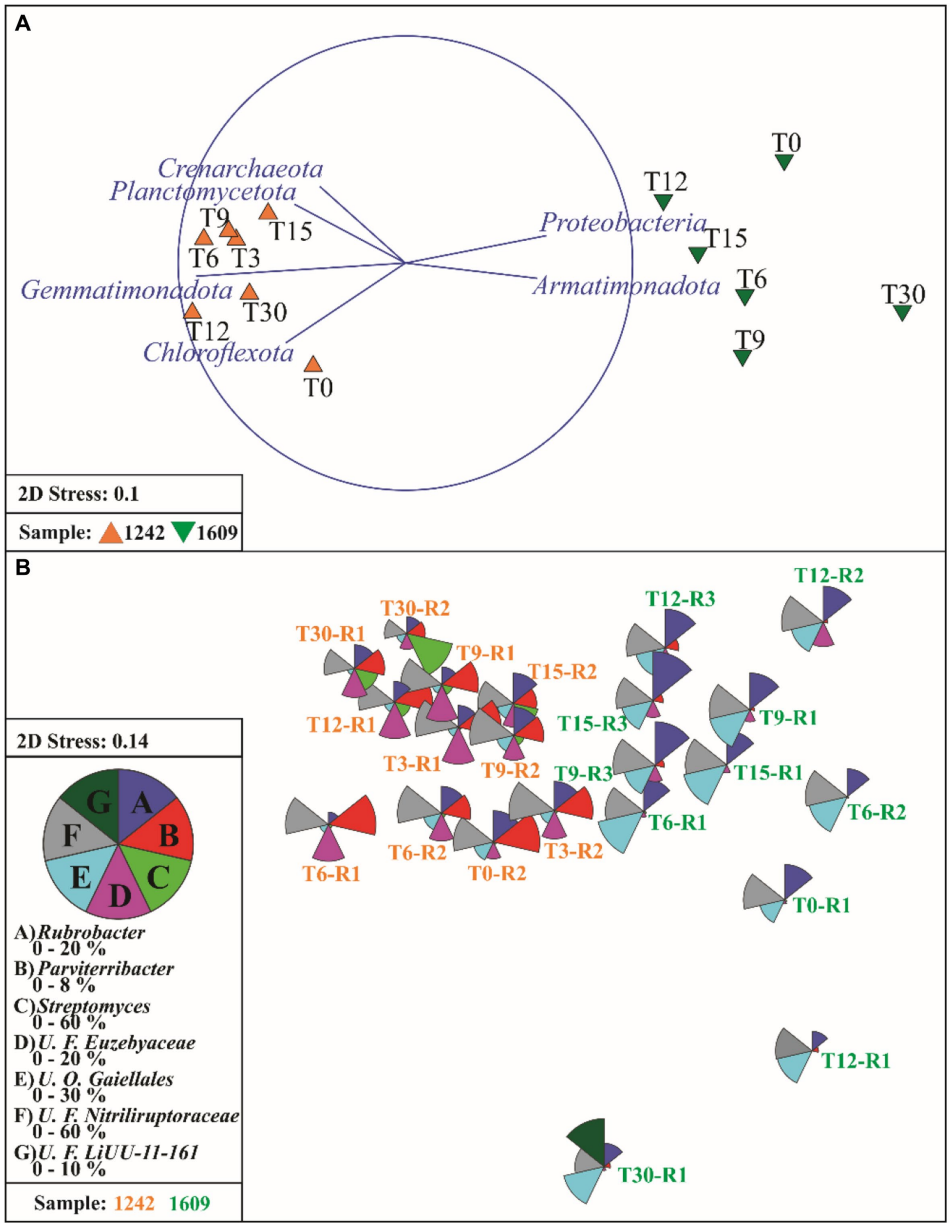
Non-metric multidimensional (NMDS) scaling of Bray-Curtis similarities for fourth root transformed phylum abundance data **(A)** and vectors of phyla which relative abundance has a Spearman correlation >0.50 with ordination axes. **(B)** NMDS plot of samples using the fourth root-transformed microbial genera abundance data, overlaid with the segmented bubble plot showing the percentage contribution of seven selected genera from *Actinobacteriota* phylum (*Streptomyces*, *Rubrobacter*, *Parviterribacter*, unclassified Families *Nitriliruptoracea* and *Euzebyaceae*, unclassified Order *Gaillales*) and one selected from *Bacteriodota, U. F. LiUU-11-161,* to the whole community. T0 to T30 indicates the incubation time in days. R1 to R3 indicates the replicate.

The site factor explained a larger proportion of the variation in microbial communities’ dissimilarity patterns (PERMANOVA site value of *p* < 0.005, *R*^2^ = 0.55). Six phyla were most strongly correlated with the different similarity patterns between the two soil microbial communities (BEST rho = 0.997), Actinobacteriota, Gemmatimonadota, Chloroflexota, Crenarchaeota, Proteobacteria (now Pseudomonadota), and Bacteroidota. The ordination analysis (NMDS) of all data (before and after wetting) revealed two well-defined groups (Figure 4A). The relative abundances of four main bacterial phyla (Actinobacteriota, Gemmatimonadota, Chloroflexota, and Proteobacteria) explained the variation between the two groups (Spearman correlation over 0.50). The main difference between the groups is a higher abundance of Gemmatimonadota (average 3.73% vs. 0.13%), Chloroflexota (average 4.36% vs. 3.21%) and Actinobacteriota (average 86.70 vs. 84.27) in YUN1242 than in YUN1609 and a higher abundance of Proteobacteria (average 7.47 vs. 2.78), in YUN1609 than in YUN1242 (Figure 4A).

The distribution (NMDS) of the most abundant actinobacterial orders revealed the unique composition of the YUN1242 and YUN1609 (PERMANOVA site value of *p* < 0.005, *R*^2^ = 0.39). Significant contributions to the differences in site community composition (Spearman’s correlation >0.75) were explained by 0319-7L14, and *Gaiellales* for YUN1609 and by *Streptomycetales*, *Euzebyales*, and *Pseudonocardiales* for YUN1242 (Supplementary Figure S8).

Ordination analysis of the distribution of the ASVs from the most abundant actinobacterial genera revealed the distinct response to soil wetting of the YUN1242 and YUN1609. Figures 4B, 5A,B shows the temporal increase of *Streptomyces* and concurrent decrease of unclassified family *Nitriliruptoraceae*, in the community of the YUN1242 experiment, and the stability of the community up to almost the end of the experiment in YUN1609 (see also Figure 3B).

A temporal analysis of the relative abundance of key ASVs assigned to each taxon showed the differences in the response of taxa from both sites to wetting. The low DNA concentrations retrieved from the replicate soil samples limited the yield of quality sequences from every time point and every flask. As shown in Supplementary Table S4B, the experiment generated from 0 to 3 data points from each site for each time point. Therefore, we looked for temporal changes in ASVs patterns based on a comparison of the first and second halves of the experiment (defined in Supplementary Table S6). The YUN1242 community showed a simultaneous significant increase of *Streptomyces*, *Frankiales*, and *Nocardioidaceae* (Figures 5A,C, and Supplementary Figure S9B respectively, and Supplementary Table S6), and a significant decrease of unclassified *Nitriliruptoraceae* family (Figure 5B and Supplementary Table S6) (One-way ANOVA value of *p* < 0.01), and *Parviterribacter* from *Thermoleophilia* class (Supplementary Figure S9A and Supplementary Table S6) (One-way ANOVA value of *p* < 0.05) during the 30-day incubation period. The *Streptomyces* growth was modeled as an exponential profile (*R*^2^ = 0.99) up to day 15 (Supplementary Figure S10), and it represented more than 15% (average 41%) of the total community at day 30 (Figure 3B). Other abundant taxa, like *Rubrobacter* did not show significant changes for either soil community (Supplementary Figure S11A). In the YUN1609 community, a significant increase was observed in the relative abundance of *Nocardioidaceae* (from Actinobacteriota phylum) during the wetting experiment (Figure 5C). In addition, *Sphingobacteriales*, *Burkholderiales* and *Ferrovibrionales* showed an exponential increase after 15-day incubation (Figure 5D), but we were not able to collect enough data to statistically test the significance of that increase because sufficient sequence reads for analysis (> 1,500) were retrieved from just one of the three T30 flasks for the YUN1609 sample. Similarly, the increase of *Pseudonocardiales*, mainly represented by *Pseudonocardia nigra* previously isolated from AD soils (Trujillo et al., 2017), was evidenced in YUN1242 sample in only one of the two replicate flasks (data not shown).

### Differences between replicated experiments

Of additional interest were observed differences in the relative abundance patterns of specific phylotypes in replicates of the same treatment during the incubation experiment, i.e., (i) the relative abundance pattern of *Rubrobacter* (i.e., in ASV4) was significantly different (value of *p* < 0.05) in replicated flasks from YUN1242 experiment (Supplementary Figure S11A); (ii) the relative abundance profiles of *Gaillales* ASVs were significantly different (value of *p* < 0.01) in the replicated flasks (Supplementary Figure S11B and Supplementary Table S6) from the YUN1609 experiment; and (iii) a sudden increase in the relative abundance of Gemmatimonadota sequences was significantly observed in only one replicated flask of the YUN1242 experiment (Supplementary Figure S11C).

## Discussion

The literature provides evidence for the occurrence of active microbial communities in the AD soils with the capacity to respond to rain events; however, the data is based on natural events over large time periods (Schulze-Makuch et al., 2018) under complex soil environmental conditions. The results of this study facilitate a detailed temporal analysis of the microbiome response of two hyperarid AD soil communities to a simulated rainfall event in the absence of nutrient amendment. The moisture conditions approximate a natural AD rainfall event.

### General impact of site factor and soil moisture

The results confirm significant differences between the two hyperarid soil communities analyzed in this study. Initial bacterial and archaeal gene copy number and alpha diversity were significantly higher in the YUN1242 soil than YUN1609. Values for both soils were similar to previous reports from hyperarid regions of the AD; between BDL and 9.5 × 10^2^ bacterial gene copies g^−1^ soil in 2016/2017 (Schulze-Makuch et al., 2018); 10^3^ bacterial gene copies g^−1^ soil (Fletcher et al., 2011; Valdivia-Silva et al., 2016), and between 2.7 and 6.7 × 10^3^ cells g^−1^ soil estimated by DAPI (Crits-Christoph et al., 2013). Archaea were less abundant than bacteria at both sites as has been reported in other arid and hyperarid soils (7% of the total prokaryotic soil biomass) (Bar-On et al., 2018).

Multiple factors can explain the greater abundance and diversity of bacteria and archaea at YUN1242 relative to YUN1609. Air RH was higher at the YUN1242 site (Table 1), a condition that can impact soil moisture levels under extreme hyperaridity (Neilson et al., 2017; Schulze-Makuch et al., 2018). In addition, higher NaCl content in the YUN1242 surface soils (Supplementary Figure S1) may contribute to the higher water content measured at this site (Vitek et al., 2010; Neilson et al., 2017; Lee et al., 2018).

Finally, soil microbial community composition in the AD was previously found to be strongly correlated with soil electrical conductivity (EC) and historically the YUN1242 EC was four times higher than that of YUN1609 (Neilson et al., 2017). The difference observed between the EC previously reported from 2015 and 2017 YUN1242 samples could be assigned to soluble salts downward remobilization typically evidenced in AD soil profiles (Ericksen, 1981; Rech et al., 2003; Voigt et al., 2020). We hypothesized that today’s microbial community correlate more to the history of high EC (2015) (Neilson et al., 2017), potentially associated to a more productive event, than the current (2017) (Table 1) EC level.

Following wetting, a significant increase in bacterial copy number was observed for both YUN1242 and YUN1609 soils (value of *p* < 0.02). The qPCR data confirm the presence of viable and metabolically active taxa in both soil microbial communities with the capacity for replication in response to simulated rainfall. In contrast, the archaeal gene copy number decreased in soils from both sites (Figure 2B). These results indicate that bacteria from the hyperarid, nutrient-poor soils of the AD are resilient and can have a growth response to wetting events that occur even in the absence of added nutrients. In contrast, the response of the archaea suggests lower resilience which is consistent with previous findings on the impact of aridity on archaea relative abundance (Neilson et al., 2017).

### General microbial community functional capacity

The structure of the microbial communities at the phylum level remained stable after hydration (Figure 3). This response is distinct from that observed with biocrusts from a hyperarid region in the Negev Desert, where a drastic collapse of *Actinobacteriota* occurred (Angel and Conrad, 2013; Baubin et al., 2022). Despite community stability at the phylum level, dynamic, but distinct shifts were observed within the actinobacterial community for both the YUN1242 and YUN1609 soil microbiomes. Further, more than 12% of the ASVs present at day 15 were not detected in the T0 communities of either site. Previous research has shown that both dead and living microorganisms are present in the hyperarid soils of the AD as indicated by measurable extracellular DNA (eDNA) and intercellular DNA (iDNA), respectively (Schulze-Makuch et al., 2018). Thus, we assume that a significant portion of the T0 ASVs were associated with eDNA or dead microorganisms. This group represents an important component of the soil organic matter available as nutrients for the survival and growth of viable microbial populations. The analysis of fluctuations in relative abundances of specific phylotypes (growing, persisting, or declining) during the 30-days of the wetting experiment targets the viable and metabolically active ASVs in the microbial communities and provides predictions for the functional capacities and survival mechanisms of microbial communities colonizing hyperarid soils of the AD.

Both soil microbiomes included specialist taxa adapted to survival in saline and dry environments. Adaptations include the production of: (1) endogenous compatible solutes by *Nitriliruptoraceae* (Chen et al., 2020) that include small organic molecules such as sugars and amino-acids, involved in cell protection against osmotic pressure and desiccation (Vargas et al., 2006); and (2) biopolymers like polyhydroxyalkanoates (PHA) by *Rubrobacter* which are considered extremolytes (Kourilova et al., 2021). PHAs not only serve as a reserve of carbon, energy, and nitrogen for cell survival during starvation (Vargas et al., 2006), but also contribute to the organic matter associated with dead cell debris (Jansson and Hofmockel, 2020). The relative abundance of *Rubrobacter* is greatest in YUN1609; however, the total bacteria abundance (16S rRNA copy number) is greater in YUN1242 so the *Rubrobacter* absolute abundance is actually providing higher levels of PHA reserves for the YUN1242 than the YUN1609 communities.

**Figure 6:**
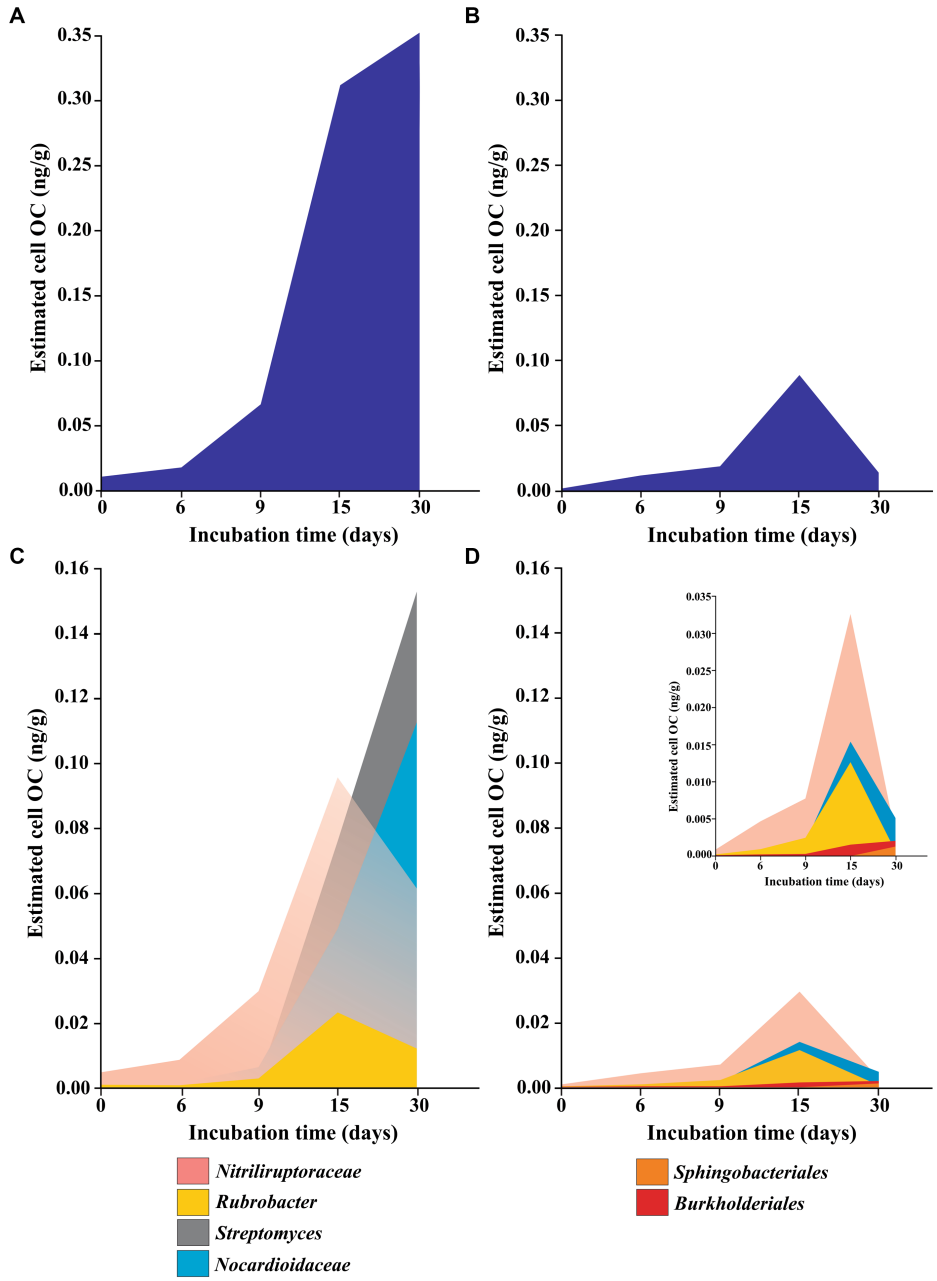
Approximation to total **(A,B)** and specific **(C,D)** cell organic carbon content (OC) of YUN1242 **(A,C)** and YUN1609 **(B,D)** estimated assuming 4.2 as the average 16S copy number per cell (Vetrovsky and Baldrian, 2013) and the relative abundance of each taxa. The insert in **(D)**, with a different Y axis scale, was included to improve the understanding. Only the main taxa responsible for microbial community dynamics were included.

Results showed a decrease in *Nitriliruptoriales* relative abundance in both communities beginning at T12 for YUN1242 and by T30 for YUN1609. This pattern demonstrates a common feature of desert soils in which the growth of one microbial group comes at the expense of the cell death of other members of the community (Angel and Conrad, 2013). Despite the observed decrease in *Nitriliruptoriales* relative abundance in YUN1242, the total abundance actually plateaued (Figure 6) due to the overall increase in the total bacterial cell number (qPCR analysis of 16S rRNA bacterial copy number). In contrast, the total abundance of *Nitriliruptoriales* decreased after T15 for YUN1609. It is likely that *Nitriliruptoriales* was less competitive in the respective communities due to a lower affinity for OC relative to the other oligotrophic taxa within the community (Figure 6). The compatible solutes produced by *Nitriliruptorales* provide more easily degraded OC for the community than the PHA, produced by *Rubrobacter*, whose degradation potential has been reported by a dozen phyla (Viljakainen and Hug, 2021; Figure 6).

In contrast, *Rubrobater* was more persistent in both communities after wetting. This is potentially due to the ability of this taxon to store carbon in PHAs (Kourilova et al., 2021) and to its reported ability to co-assimilate organic and inorganic carbon through a mixotrophic growth strategy. *Rubrobacter* uses the acetyl-CoA pathway (Leon-Sobrino et al., 2019) supported by available energy resources in desert soils such as light and hydrogen (Meier et al., 2021). In addition, the *Rubrobacter* genus has been described as a “CO tolerant capnophilic, nitrogen scavenging oligotroph” (Norman et al., 2017). Taxa from the *Rubrobacterales* order are considered active members of the Yungay soils microbial communities based on their identification in the iDNA fraction (Schulze-Makuch et al., 2018), and in the Namib desert where that family represented 7% of the active microbial community (Leon-Sobrino et al., 2019). Despite their resilience, a decrease in relative abundance was observed at the end of this experiment in YUN1609.

Increases in the *Nocardioidaceae* family were observed in both communities after T9. Members of this oligotrophic and motile family *Marmoricola* and *Marmoricola* like (Bartelme et al., 2020; Zhang et al., 2021) produce extracellular enzymes mainly involved in N-recycling such as aminopeptidases. Not only that but *Marmoricola* are also characterized by a higher affinity for OC (low *Km* for organic molecule transport), lower energy requirement for cell maintenance, and low respiration activity (Semenov, 1991). In addition, *Nocardioidaceae* along with other taxa present in the YUN1242 community following soil wetting (*Streptomyces, Frankiales*, and *Pseudonacardia*) are characterized by sporulation and mycelia production. Taken together these microbial properties are important because prior to wetting, soil dryness and heterogeneity create isolated conditions (Neilson et al., 2017; Petrenko et al., 2020) limiting symbiotic, commensalistic, and competitive interactions between taxa. Immediately after wetting, the resulting water films facilitate spore germination and mycelia formation, microbe-microbe interactions and create greater potential essential shared resource utilization within the microbial communities. Soil wetting can enhance the production of these extracellular enzymes to improve the utilization of OC available following the cell death of other microorganisms. Similar increases in the abundance of soil Actinobacteriota capable of producing aminopeptidase enzymes involved in N-cycling under drought (i.e., *Streptomyces*, *Marmoricola*, and other *Nocardioides*) have been previously reported (Zhang et al., 2021).

### Specific functional potential of the YUN1242 microbiome

The initial YUN1242 community composition differed from YUN1609 by a greater relative abundance of Gemmatimonadota and Choroflexota phyla (Figures 34A). In addition, within the Actinobacteriota phylum, YUN1242 was characterized by a greater relative abundance of *Euzebyales* order (*Nitriliruptoria* class, Actinobacteriota) (Figure 3B). Interestingly, both *Euzebyales* and *Nitriliruptorales* are specialized to survive in saline environments (Neilson et al., 2017; Yin et al., 2018), however, unlike *Nitriliruptorales*, the *Euzebyales* relative abundance was sustained throughout the wetting experiment. The higher relative abundance of these taxa in YUN1242 is logical, considering the history of higher Cl^−^ and EC levels observed at this site relative to YUN1609 (Neilson et al., 2017).

After hydration, significant changes were observed in the YUN1242 microbial community within Actinobacteriota (Supplementary Figures S7, S9, S11). Increases in the relative abundances of *Streptomyces* and *Frankiales* were observed beginning at T9 and T12, respectively, with concurrent decreases in the relative abundances of *Nitriliruptorales* (initial relative abundance 37.8%) and *Parviterribacter* (initial relative abundance 4.2%). These results resemble what was previously observed in culturing experiments with AD hyperarid soils (Schulze-Makuch et al., 2018). *Parviterribacter* is reported to have a saprophytic lifestyle based on their preference for complex proteinaceous substrates and glucose as carbon sources observed in the strains isolated from Namibian desert soil (Foesel et al., 2016). Thus, this phylotype may have thrived during more active episodes following precipitation events. Cell death of *Nitriliruptorales* and *Parviterribacter* provides OC for the growth of *Streptomyces* and *Frankiales* during the experiment (Figures 4B, 5A and Supplementary Figure S9B). A recent calculation revealed that soil microbial biomass accounts for more than half the OC in soils from agricultural, grassland, and forest ecosystems (Liang et al., 2019), and it is hypothesized that microbial biomass comprises an even greater percentage of the OC reserves in deserts (Azua-Bustos et al., 2017).

The results suggest a mixotrophic growth strategy for *Streptomyces* and *Frankiales* within this hyperarid microbial community. The cell growth observed (Figure 2) in YUN1242 soils suggests an estimated 13-fold increase in OC content by T6 to T9 [an approximation of the cell OC content was estimated by the reported OC content per cell, 26.02 ± 1.08 fg C cell^−1^ (Troussellier et al., 1997), and the 16S rRNA gene copy numbers determined by qPCR, assuming the occurrence of 4.2 16S rRNA gene copy per cell in all the taxa based on the average number reported (Vetrovsky and Baldrian, 2013; Figures 2, 6)]. The mixotrophy strategy combines heterotrophy (supplied by OC from microbial cell death) and autotrophy (chemolithotrophy, phototrophy) (Stoecker et al., 2017) for carbon acquisition when OC supplies are limited.

Obligate and facultative CO and H_2_-supported autotrophic metabolisms have been confirmed in the *Streptomyces* genus (Kim and Goodfellow, 2002). Threshold values of 0.2 μL L^−1^ of CO were estimated for *S. thermoautotrophicus* growth which is comparable to levels observed in various soils (Gadkari et al., 1990). The appearance of *Streptomyces* at T9 (0 to >30% of relative abundance) (Figures 3B, 5A) was potentially supported by the occurrence of carboxydotrophy/hydrogenotrophy in YUN1242. Reduced growth and cell death of *Nitriliruptorales* probably resulted from exhaustion of intracellular OC reserves and the inability to compete with the (Troussellier et al., 1997) faster growth of *Streptomyces* based on its mixotrophic growth strategy. An H_2_-supported growth capacity previously reported for *Frankia* (Sellstedt and Richau, 2013) could also allow this phylotype to contribute to the mixotrophic metabolism of the microbial community, and explain the increase in *Frankia* relative abundance in the YUN1242 wetting experiment.

Nitrate reduction has been suggested as the main source of available N_2_ in desert soils (Leon-Sobrino et al., 2019). Several members of the obligate aerobic genus *Streptomyces* can reduce nitrate (Fischer et al., 2014) which could allow *Streptomyces* to couple CO oxidation to nitrate reduction to nitrite (dissimilatory nitrate reduction) or dinitrogen (denitrification) (King and Weber, 2007; He et al., 2021). The capacity of *Frankia* for fixing N_2_ suggests a syntrophic association in the proliferation of these two phylotypes. In addition, the Frankia genus demonstrates uptake hydrogenase activity (Tisa et al., 2016) that facilitates the recycling of the H_2_ produced by nitrogenase during N_2_ fixation, thus improving the efficiency of N_2_ fixation (Gtari et al., 2012, 2019). H_2_ oxidation provides energy (ATP), reducing equivalents, and removes O_2_ which improves nitrogenase activity (Tamagnini et al., 2002). Thus, we propose a syntrophic association between *Streptomyces* growth respiring nitrate and *Frankia* N_2_ fixation, and H_2_ production.

The Gemmatimonadota and Choroflexota phyla add to the metabolic potential of the YUN1242 microbiome. Gemmatimonadota are associated with multiple assimilative and dissimilative N processes, including the ability to perform the terminal step in denitrification by NO_2_ removal (Chee-Sanford et al., 2019). In addition, *Longimicrobiacea* (Gemmatimonadota) is an oligotrophic microorganism capable of storing intracellular polyphosphate granules, a potential phosphorous source for the growing community. This taxon has also been added to the list of bacterial phyla containing anoxygenic phototrophic species (Bull et al., 2018; Koblizek et al., 2020; Mujakic et al., 2021; Zeng et al., 2021). Chloroflexota (Figure 3) includes *Thermobaculum*, a non-phototrophic gram-positive, heterotrophic, thermophile (Botero et al., 2004; Kuhlman et al., 2006; Kiss et al., 2010; Kunisawa, 2011; Gupta et al., 2013). This phylotype has the potential to use CO and H_2_ as energy sources for growth based on evidence from one of its closest relatives, *Thermomicrobium roseum*, that can persist mixotrophically on atmospheric gases (Wu et al., 2009; Houghton et al., 2015; Islam et al., 2019). Chloroflexota in general have a wide distribution of enzymes responsible for mixotrophic metabolisms using reduced gasses (Islam et al., 2019) and they have been reported to colonize new soils after volcanic eruptions (Hernandez et al., 2020; Sterling et al., 2022).

### Specific metabolic potential of the YUN1609 soil microbiome

The YUN1609 soil microbial community was characterized by significantly lower T0 microbial biomass than YUN1242 (lower total bacterial and archaeal abundance). YUN1609 had a greater and more significant relative abundance of *Gaiellales* relative to YUN1242 (Figures 3B, 4B). From T0 to T15, carbon fixation is suggested to play a relevant role in the microbial community persistence and growth based on the CO_2_ fixation capacity predicted by the genome analysis of the *Gaiella occulta* and the growth of marine isolates from this taxon in an inorganic medium (Chen et al., 2021). Nitrate reduction was also evidenced by culturing the type strain (Severino et al., 2019; Chen et al., 2021; Ai et al., 2022). The greater relative abundance of *Gaiellales* in the YUN1609 community relative to YUN1242 could be explained by the low salt tolerance of the type strain *Gaiella occulta* (Albuquerque et al., 2011). YUN1609 soils have a history of lower Cl^−^ and EC levels than YUN1242.

Furthermore, nitrate reduction was previously predicted for two taxa with higher abundance in YUN1609, the Unclassified Class 0319-7L14 (Zhang et al., 2019) and the Family 67–14 (Kantor et al., 2017). The Unclassified Class 0319-7L14 isolated from Australian arid soils, was reported in an arid soil ecosystem in China, and in semiarid, unseeded control sites during mine waste revegetation in the US (Shange et al., 2012; Zhang et al., 2019; Ossanna et al., 2023). The Family 67–14 was isolated from a thiocyanate stock bioreactor (Kantor et al., 2017).

After T15, the appearance of new taxa outside Actinobacteriota phylum was observed. These included the Unclassified LiUU-11-161 (Eiler and Bertilsson, 2004) (*Sphingobateriales*/Bacteroidota), *Zoogloeae* (Rossello-Mora et al., 1995), uncultured *Commamonadaceae*, *Rhodoferax* genera (*Burkholderiales*), and *Ferrovibrio* (*Ferrovibrionales*/*Alphaproteobacteria*) (Dahal and Kim, 2018; Figure 3). A simultaneous decrease in *Nitriliruptorales* (Figure 3) relative abundance and in overall estimated viable cell OC was observed.

*Rhodoferax* and *Zoogloea* genera, are capable of carbon and N_2_ fixation, respectively. Anaerobic, photoheterotrophic or photoautotrophic (using H_2_ or reduced sulfur compounds [RSC]), aerobic heterotrophic, anaerobic fermenter, N_2_ fixation (Willems, 2013), iron-reducing and microaerobic Fe(II) oxidation (Kato and Ohkuma, 2021) metabolisms, have been evidenced in *Rhodoferax* genus (Comamonadaceae/Burkholderiales). Capacity for nitrate reduction and N_2_ fixation was reported in *Zoogloea* genus from *Rhodocyclaceae* family (Dahal et al., 2020). In addition, *Zoogloea* sp. N299 was described as an aerobic oligotrophic denitrifier isolated from autotrophic nitrate removal reactors (Huang et al., 2015). Nitrate reduction was reported as well in *Ferrovibrio* genus (Dahal and Kim, 2018). Furthermore, a key role in reductive sulfate assimilation and cycling has been also suggested for *Burkholderiales* in soil desert environments (Leon-Sobrino et al., 2019).

We argue that the proposed low level of carbon fixation activity sustained during the first 15 days of the experiment by *Gaillales* and *Rubrobacter* provided carbon reserves for the increase in the relative abundance of new taxa at the end of the experiment (*Nocardiodaceae*, *Zoogloea*, *Rhodoferax and Ferrivibionales*). The decrease in *Nitriliruptoraceae* provided potential OC reserves as explained for YUN1242. The new phylotypes increased the proposed functional capacity of this microbiome for nitrate reduction in aerobic/microaerobic conditions, nitrogen fixation, and iron oxidation/reduction. These slow-growing oligotrophic microorganisms that can use inorganic sources of energy appear to be able to compete for the scarce OC at the end of the experiment. However, the proposed mixotrophic metabolic capacity that evolved in the YUN1242 microbiome in response to soil wetting was not observed in this community. We maintain that the 4-fold lower microbial biomass content as estimated by 16S rRNA gene copy number at T0 may explain this difference in metabolic capacity.

It is important to note that some potential conclusions from this research were limited by the difficulties in obtaining sufficient DNA from all replicates at each time point and the significant differences observed between replicates at a few time points. The variation observed between replicate flasks could be explained by the occurrence of isolated assemblages in the hyperarid environments (Neilson et al., 2012) which were probably due to the restricted motility expected for microorganisms in soils of low a_w_ (Kim and Goodfellow, 2002; Jones et al., 2018). The results obtained from this research will guide future experiments to characterize the distinct metabolic capacities of these two communities.

## Conclusion

The erratic precipitation events that control the metabolic activity of desert soil microbiomes are extremely rare in the core region of the AD. The results from this controlled, temporal analysis demonstrated the presence of viable cells in AD hyperarid soils with sufficient resources to support growth following a precipitation event despite such extended periods of desiccation. Thirty-day changes in the bacterial community composition of the studied sites revealed two distinct predicted strategies for survival. Initial bacterial and archaeal abundance and their associated nutrient reserves were significantly greater in the YUN1242 community. The YUN1242 microbiome evolved quickly from a community dominated by taxa that can accumulate resources to survive (*Nitriliruptoraceae*, *Parviterribacter*, and *Rubrobacter*) to one dominated by spore-forming, mixotrophic taxa able to use microbial biomass, and atmospheric gases to fix CO_2_, and N_2_, with O_2_ and nitrate used as electron acceptors. In contrast, slow biomass accumulation of facultative autotrophic taxa characterized growth in the drier YUN1609 community. The proposed syntrophic association between Streptomyces growth respiring nitrate and Frankia N_2_ fixation, and H_2_ production in the YUN1242 suggests a capacity for critical microbe-microbe associations in this soil microbiome following a rainfall event. The results indicate that slight differences in available moisture for extreme hyperarid soil microbial communities can have significant impacts on community composition, functional potential, and responses to soil wetting. The microbial profiles and proposed metabolic strategies, including microbial interactions, defined by this study can be used as long-term biomarkers of hyperaridity, potential biosignature distribution on other planets, and for bioprospecting novel biological capacities.

## Materials and methods

### Sample collection

We studied previously characterized surface soils of a hyperarid region in the AD (Neilson et al., 2017). Hyperarid sites YUN1242 and YUN1609 were selected from five previously characterized hyperarid sites along the transect due to their distinct microbial communities. YUN1242 and YUN1609 had average soil relative humidity values of 20.9 and 17.2%, respectively. YUN1242 is located close to the coast with exposure to coastal fogs, whereas YUN1609 is over 90 km further inland beyond the coastal fog zone. Further, distinct microbial communities were observed at the two sites in samples collected in 2012 (Neilson et al., 2017). For example, Pseudonocardiaceae (20% relative abundance) and Euzebya (11%) comprised 33% relative abundance of the YUN1242 bacterial/archaeal community, whereas the relative abundances of these taxa in YUN1609 soils were just 0.2 and 0.3%, respectively. In contrast, the YUN1609 soils were dominated by Acidimicrobiales_koll13 (45% relative abundance) and Gaiellaceae (11%); two taxa with relative abundances of just 5 and 1% in the YUN1242 community.

The samples YUN1242, and YUN1609 were collected from the west–east southern Yungay (YUN) transect, on 25th January 2017 (Figure 1). Global Positioning System (Garmin GPSMAP 60CSX) coordinates, and elevations for all sites are listed in Table 1. Using a thermo-hygrometer (Hanna Instruments Inc., United States), temperature and relative humidity parameters for each site were determined from a 50 cm deep soil pit. UVA, UVB, and UVC irradiances and photosynthetic active radiation (PAR) were measured with a portable photo-radiometer HD2302.0 (Delta OHM, Italy). In addition, three soil samples were collected, using sterilized instruments, from three different sidewalls at a 10–20 cm depth of each pit and stored in sterile transparent reclosable bags. The bags were sealed with tape, kept in a cooler and transported immediately to the lab (less than 100 km away from the site), and stored at environmental temperature (~20°C).

### Soil analysis

The bags were unsealed in the lab and gravimetric moisture content was immediately measured. Moisture contents were determined in triplicate for each site. The soil sample (10 g) was added to a glass petri dish, incubated at 105°C for 24 h, and weighed after the dish was cooled down for 2 h. The procedure was repeated to obtain a constant result (<0.1%) between two successive weights (Blaska and Fisher, 2014). Soil pH, electrical conductivity (EC), and redox potential (ORP) were determined in a 1:1 slurry of 2 mm-sieved soil in distilled water after 1 h of shaking followed by 1 h of rest by using a Hanna HI 9829 multiparameter as described before (Neilson et al., 2017).

### Wetting experiments

Immediately after having the result of the GWC of the samples (5 days after sampling), we started the experiment to determine the potential effects of wetting on both soil microbial communities. We sieved (<2 mm) 50 g of every soil sample, which were then added to sterile 250 mL flasks (Supplementary Figure S3). This procedure was repeated for each of the three soil samples collected from each site. Sterile water was introduced directly to the soil surface of the flasks until it reached 5% of moisture to simulate desert rainfall. The 5% moisture level was selected based on records of gravimetric moisture contents from desert soils following rainfall events and confirmed by samples collected from the two sample sites following the June 2017 rainfall event (Table 1). June 2017 moisture contents were 4.196 and 6.150% for sites YUN1242 and YUN1609, respectively. Afterwards, flasks were sealed with hydrophobic cotton and incubated for 30 days at 20°C under light-oxic conditions. The moisture was controlled by the measurements of weight difference at regular intervals throughout the experiment to maintain a 5% GWC considering the water retention observed in soils after rain events (Table 1; Pfeiffer et al., 2021).

Subsamples were retrieved from every flask on days 0, 3, 6, 9, 12, 15, and 30 of incubation and DNA was immediately extracted from 2 g of wet-weight soil from each sample, including a blank tube with the DNA-free reagents used, according to FastDNA SPIN kit for soil method (MPbio, United States). We quantified the DNA with a Fluorimeter (Quantus, Promega, United States), and the DNA integrity was measured by a Nanodrop NP1000 spectrophotometer (NanoDrop Technologies, United States) labeling the ratio of DNA/RNA 260/230 and the ratio of DNA/protein 260/280. The obtained concentration of all the subsamples ranged from 18 to 900 ng of DNA g-1 dry soil (Supplementary Figure S4). The extract concentration was less than 0.5 ng μL^−1^ in the control tests. Most of the extracted DNA showed acceptable quality (0.9–2.0 A 260/280).

### qPCR analysis

The abundance of Bacteria and Archaea was quantified in duplicate by quantitative-polymerase chain reaction (qPCR) using Bacteria (UniBactF336: GACTCCTACGGGAGGCAGCA, UniBactR937: TTGTGCGGGCCCCCGTCAAT) and Archaea universal primers for 16S rRNA gene (ARC344F: ACGGGGNGCANCAGGCG, ARC915R: TGCTCCCCCGCCAATTCC) (Demergasso et al., 2010). The correlation coefficient for the standard curves was 0.99 and the PCR efficiency was on average 93%. In each periodic DNA extraction, control was also performed from the Kit reagents. A Rotor-Gene Q Real-Time Cycler (Qiagen, United States) was used for qPCR reactions and the data was processed using its software. Each reaction was 10 μL in volume and contained the following mixture: 5 μL of SensiMix SYBR No-Rox Kit (Bioline, United Kingdom), 2 μL nuclease-free water, 1 μL of the corresponding oligonucleotide primer (0.5 μM), and 1 μL of the template. The bacterial amplification program consisted of 40 cycles of 95°C for 30 s, 65°C for 30 s, and 72°C for 20 s while the archaeal program consisted of 40 cycles of 95°C for 40 s, 65°C for 40 s, and 72°C for 20 s. 16S rRNA gene copy number per g of each subsample was determined, as reported previously (Remonsellez et al., 2009), using the equation:


$$\mathrm{copies}/g=\left(\left(\mathrm{copies}/\mathrm{\mu l}\right)*100*\mathrm{dilution}\right)/g\;\mathrm{of}\;\mathrm{initial}\;\mathrm{wet}\;\mathrm{weight}$$

In addition, DNA was used for high-throughput sequencing.

That number of copies was considered equivalent to 4.2 times the number of cells [assuming 4.2 16S rRNA gene copies per cell (Vetrovsky and Baldrian, 2013) and was used for estimating the specific cell number of each taxon (taxon relative abundance [%] * total bacterial cell numbers), and the total and specific microbial OC (total bacterial + archaeal cell numbers * OC content per cell, and specific cell numbers * OC content per cell, respectively)]. We used 26.02 ± 1.08 fg C as the OC content per cell (Troussellier et al., 1997).

### 16S rRNA sequencing

Sequencing was performed with 42 subsamples (triplicates of the 7 time points during incubation of both soil pits) and 7 blanks (DNA extraction reagents) as well as 6 controls (sequencing reagents) according to the following procedure. The hypervariable V4 region of the 16S rRNA gene was amplified from each sample using unique for each sample barcoded reverse primers (806R: GTGYCAGCMGCCGCGGTAA) and a common forward primer (515F: GGACTACNVGGGTWTCTAAT). Both the reverse and the forward primers were extended with the sequencing primer pads, linkers, and Illumina adapters (Caporaso et al., 2012). The PCR was performed using MyFi™ Mix (Bioline Meridian, Cat. No. BIO-25050) on LightCycler 96 (Roche) in the final volume of 40 μL. Amplicons were quantified using the Quant-It PicoGreen dsDNA Assay kit (ThermoFisher Scientific, Cat. No. P7589), according to the manufacturer’s protocol. Equal amounts of amplified DNA (120 ng) from each sample were pooled into a sequencing library followed by removing DNA fragments smaller than 120 bp (unused primers and dimer primers) with UltraClean PCR Clean-Up Kit (MoBio, Cat. No. 12500). The final amplicon concentration was quantified by qPCR with KAPA Library Quantification Kit for Illumina Platforms (KAPA Biosystems, Cat. No. KK4854) in the presence of the set of six DNA standards (KAPA Biosystems, Cat. No. KK4905). Subsequently, the library was diluted to a concentration of 4 nM, and denatured with 0.1 N NaOH. The library was sequenced at the Microbiome Core at the Steele Children’s Research Center, University of Arizona, using the MiSeq platform (Illumina) and custom primers (Caporaso et al., 2012). Due to the limited sequence diversity among 16S rRNA amplicons, 5% of the PhiX Sequencing Control V3 (Illumina, Cat. No. FC-110-3001) was used to spike the library to increase diversity. The raw sequencing data were demultiplexed and barcodes trimmed using *idemp* script.^1^

The accuracy of microbial community surveys based on universal marker genes suffers from the presence of contaminant DNA sequences not truly present in the sample that can come from various sources, including reagents. Contaminant ASVs were identified using a nested-PCR approach as described in the Supplementary Information Box 1. Low levels of contaminant sequences from 2 to 113 ASV counts per sample were detected which represented an average of less than 1% of abundance in target samples (Supplementary Information Box 1).

### Taxonomic and phylogenetic analysis

Demultiplexed fastq files were received and subjected to primer removal, filtered by sequence quality (for keeping Ph quality over 30, so paired reads are formed with 253 bp), denoised, merged, and chimaera removal using the DADA2 pipeline (Callahan et al., 2016). All filtered-merged sequences were assigned to amplicon sequence variants (ASV) by the DADA2 pipeline. The representative reads were mapped to the SILVA database (release 138) (Quast et al., 2013). Then we used the Phyloseq (version 1.42.0) pipeline to (a) eliminate taxa with one read, (b) remove taxa with less than 0.005% mean relative abundance across all read counts, (c) eliminate samples having less than 1,000 reads, and (d) remove the 14 bacterial sequences (n° of ASV 9, 20, 23, 19, 2, 18, 38, 29, 57, 58, 69, 72, 167, and 178) found in controls and blanks having less than, on average, 1% of abundance in targeted samples (Supplementary Data Box 1). We detected from 1,075 to 37,811 reads per sample for YUN1242 and 132 to 21,505 reads per sample for YUN1609 (Supplementary Table S3). We calculated histograms and rarefactions curves to standardize the sequence number using the rarefy_even_depth function from the Phyloseq package to standardize the sequence number. Most sequences were distributed around 1,500–5,000 reads counts (Supplementary Figure S12A) and we proceeded to rarefy the data to the smaller sample size of 1,500 reads (Supplementary Figure S12B). Finally, the read number by sequence, taxonomy table, and categorical variables (called sites, replicates, and days) associated with the samples were integrated and kept in a Primer-7 software package [Plymouth Marine Laboratory, Plymouth, United Kingdom (Clarke and Gorley, 2015)]. The ASV diagram was made using the website^2^ previously described (Oliveros, 2007–2015).

The diversity (Shannon, H′), and richness (Margalef) indices were also calculated using the measures included in the PRIMER-7 software package based on the relatedness of the species within a sample.

### Statistical analysis

Soil bacterial and archaeal total abundance (copies·g^−1^ of soil) were modeled as a function of the sampling site (YUN1242 and YUN1609) and incubation time (T0, T3, T6, T9, T12, T15, and T30) to determine significant differences between the levels of these factors (Supplementary Table S7). Modeling was carried out by using two-way repeated measures ANOVA. A log 10 transformation was used to meet ANOVA’s assumptions. In the multiple testing, value of *p* correction via the Benjamini–Hochberg false discovery rate (FDR) was performed. Model fitting and pairwise multiple comparisons were performed using the statistical software R 3.6.0. (R Core Team, 2018) and car, tidyverse and rstatix packages.

In order to compare Shannon and Margalef indices between both samples (including all the subsamples obtained from a single site during the incubation time in one group), a box plot was created, and a *t*-test was applied using KaleidaGraph version 4.5.4 for Windows, Synergy Software, Reading, PA, United States.^3^ Simple linear regression was used to fit the Margalef index distribution among incubation days for both samples (confidence bands were also calculated). Multiple linear regression was used to assess the difference in the slopes throughout an interaction term. The abundance of Amplicon Sequence Variances (ASVs) in different samples and at incubation time (days) was analyzed using Primer-7 (Primer -E) software (Clarke and Gorley, 2015). We used the fourth root transformation to homogenize the amounts of ASVs and thus reduce the dominance effect. A similarity matrix (resemblance) was constructed using the Bray-Curtis method (Bray and Curtis, 1957) included in Primer V7 software (Clarke and Gorley, 2015). We used non-metric multidimensional scaling (NMDS), included in Primer-7, to build a restricted arrangement of the ASVs or groups of other taxonomic levels based on the experiment design: (i) phylum abundance data; (ii) abundance of actinobacterial orders; (iii) microbial genera abundance data, overlaid with the segmented bubble plot (Clarke and Gorley, 2015) showing the percentage contribution of selected genera.

We performed a significance test using the permutational multivariate analysis of variance (PERMANOVA function) (Anderson, 2001) when Bray Curtis dissimilarity was tested with 10,000 permutations. Two factors were evaluated: Site (YUN1242 and YUN1609) and incubation time (0, 3, 6, 9, 12, 15, 30 days).

In addition, we analyzed the dynamics of the relative abundance of specific taxa during the wetting experiment. We performed one-way ANOVAs to evaluate the significance of the changes regarding the three identified factors, incubation time, sites, and replicates (flasks).

## Data availability statement

The raw sequence data presented in the study are deposited in the DNA Data Bank of Japan (DDBJ) repository, accession numbers DRR465014 to DRR465087.

## Author contributions

CD supervised the project and wrote the manuscript, aided by JN and CT-C. CD and JN conceived and designed the experiments. CD and CT-C carried out the field trips. Under the supervision of CD, CT-C sampled and monitored the wetting experiments, extracted DNA, and quantified the microbial abundance by qPCR. DA and CD did the statistical analysis of qPCR determination, aided by CT-C. DL did the sequencing. RV and CT-C performed the taxonomic and phylogenetic analyses. DA and CD did the statistical analysis of microbial diversity. All authors contributed to the review, editing, and revision of the article.

## Footnotes

^1^ https://github.com/yhwu/idemp

^2^ https://bioinfogp.cnb.csic.es/tools/venny/

^3^ www.synergy.com
