# Corrigendum: Hyperarid soil microbial community response to simulated rainfall

**DOI:** 10.3389/fmicb.2023.1327998

**Published:** 2023-12-01

**Authors:** Cecilia Demergasso, Julia W. Neilson, Cinthya Tebes-Cayo, Roberto Véliz, Diego Ayma, Daniel Laubitz, Albert Barberán, Guillermo Chong-Díaz, Raina M. Maier

**Affiliations:** ^1^Biotechnology Center “Profesor Alberto Ruíz”, Universidad Católica del Norte, Antofagasta, Chile; ^2^Department of Environmental Science, University of Arizona, Tucson, AZ, United States; ^3^Department of Geology, Faculty of Engineering and Geological Sciences, Universidad Católica del Norte, Antofagasta, Chile; ^4^Department of Mathematics, Faculty of Sciences, Universidad Católica del Norte, Antofagasta, Chile; ^5^Steele Steele Children's Research Center, Department of Pediatrics, University of Arizona, Tucson, AZ, United States

**Keywords:** hyperarid, soil microbiome, soil wetting, extremophiles, oligotrophic microbes, Atacama Desert, mixotroph

In the published article, there was an error regarding the affiliation for Diego Ayma. Instead of “Steele Steele Children's Research Center, Department of Pediatrics, University of Arizona, Tucson, AZ, United States,” it should be “Department of Mathematics, Faculty of Sciences, Universidad Católica del Norte, Antofagasta, Chile.”

The authors apologize for this error and state that this does not change the scientific conclusions of the article in any way. The original article has been updated.

