# Supplementary material for: Hyperarid soil microbial community response to simulated rainfall
Source: Front Microbiol. 2023 Sep 14;14:1202266. doi: 10.3389/fmicb.2023.1202266 (PMC10537920; doi:10.3389/fmicb.2023.1202266)
Supplement: Supplementary file 1 [file Presentation_1.pdf]

SUPPLEMENTARY INFORMATION

Hyperarid soil microbial community response to simulated rainfall

Demergasso C[1]*, Neilson JW[2*], Tebes-Cayo C[1,3], Véliz R[1], Ayma D[4], Laubitz D[5], Barberán A[2], Chong-Díaz G[3], Maier RM[2]

[1]Centro de Biotecnología Alberto Ruíz, Universidad Católica del Norte, Antofagasta, Chile
[2]Department of Environmental Science, University of Arizona, Tucson, Arizona, USA
[3]Department of Geology, Faculty of Engineering and Geological Sciences, Universidad Católica del Norte, Antofagasta, Chile.
[4]Department of Mathematics, Faculty of Sciences, Universidad Católica del Norte, Antofagasta, Chile
[5]Steele Children's Research Center, Department of Pediatrics, University of Arizona, Tucson, AZ United States

SUPPLEMENTARY RESULTS

A

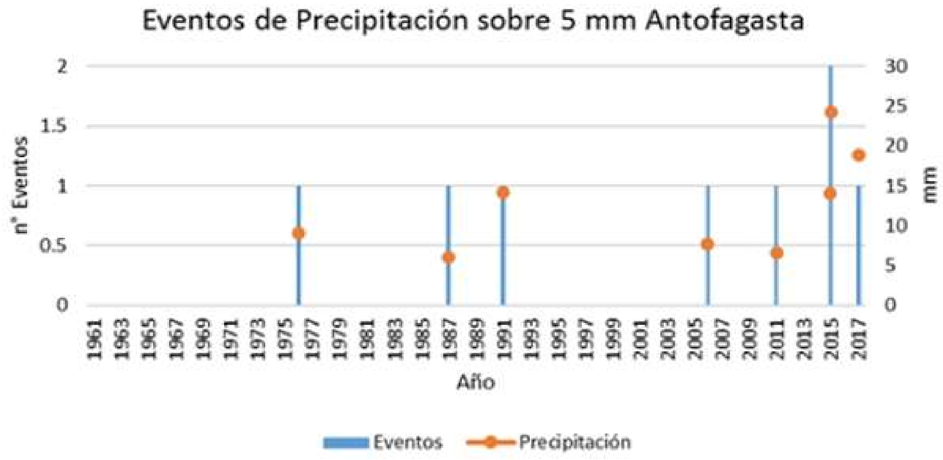

B

DATOS DEL EVENTO (7y8 de junio– hasta las 8 am)	
Nombre localidad	Precipi-tación
Lluta bajo	3.2 mm
Arica	1.0 mm
Chiu-chiu	3.2 mm
San Pedro Atacama	6.2 mm
Calama Ad.	3.0 mm
Toconao	9.6 mm
Antofagasta Aero.	19.6 mm
Antofagasta U. del	21.9 mm

Figure S1. A) Rain events (eventos in Spanish) over 5 mm at Antofagasta (the closest city to Yungay) since 1961 up to 2017. B) mm of rainfall in the rain event on June 7-8th 2017 (https://cooperativa.cl/noticias/pais/tiempo/antofagasta-soporto-el-segundo-evento-de-lluvias-mas-extremo-desde-1950/2017-06-08/131738.html).

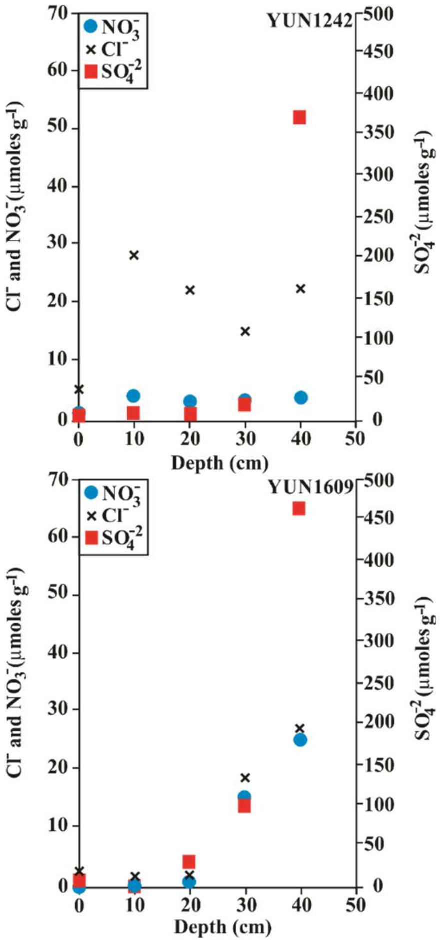

Figure S2. Observed salt abundance across a depth profile in the sites YUN1242 (above) and YUN1609 (below) selected for the study in a west-east transect (data from March 2012 [7]). Cl^-, NO_3^-, SO_4^{2-} means chloride, nitrate and sulfate ions, respectively.

Wetting soils (Test with 5% of moisture)

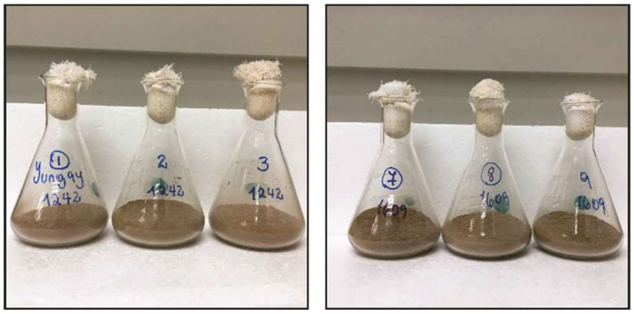

Figure S3. Picture of the replicated flasks of both sites included in the wetting experiment set up.

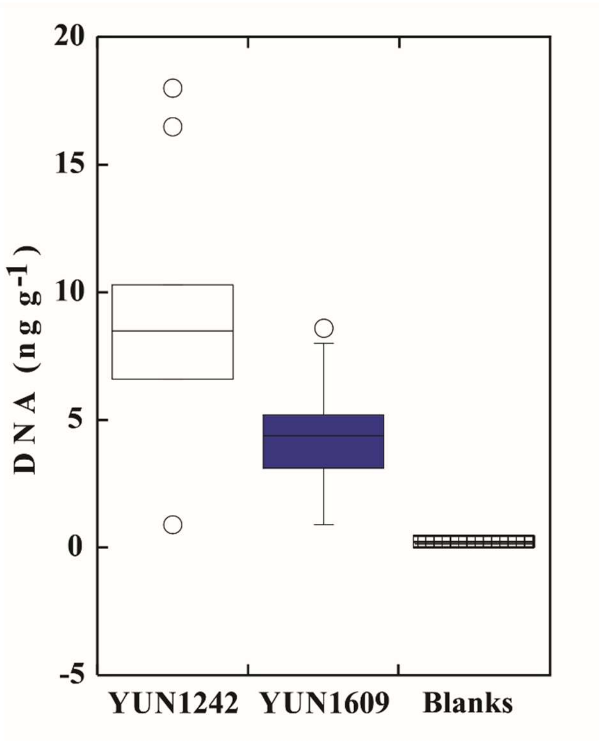

Figure S4. DNA concentration retrieved from the experiments with both soil samples and the blanks test (only reagents from nucleic acid extraction) measured by a fluorometer.

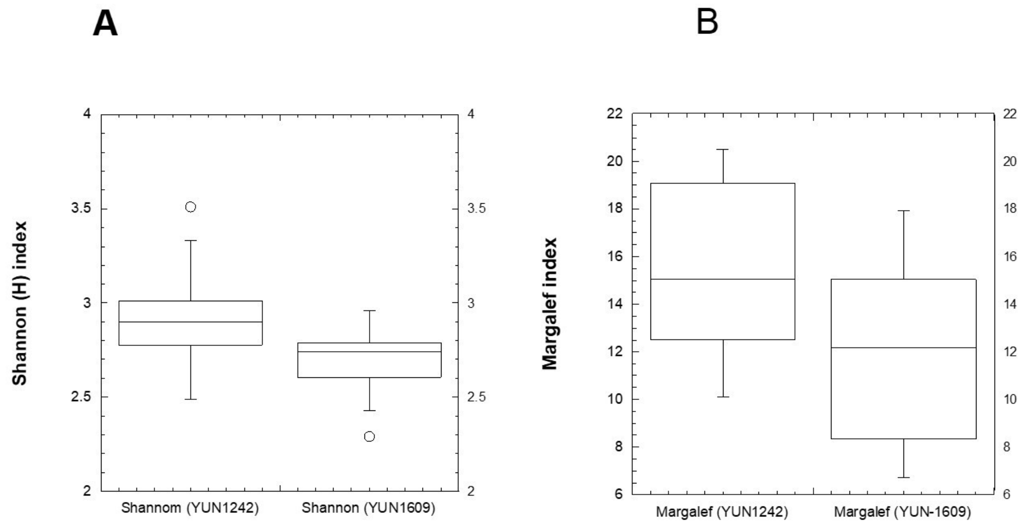

Figure S5. Diversity Shannon, H', and richness Margalef (B) indices of YUN1242 and YUN1609 samples.

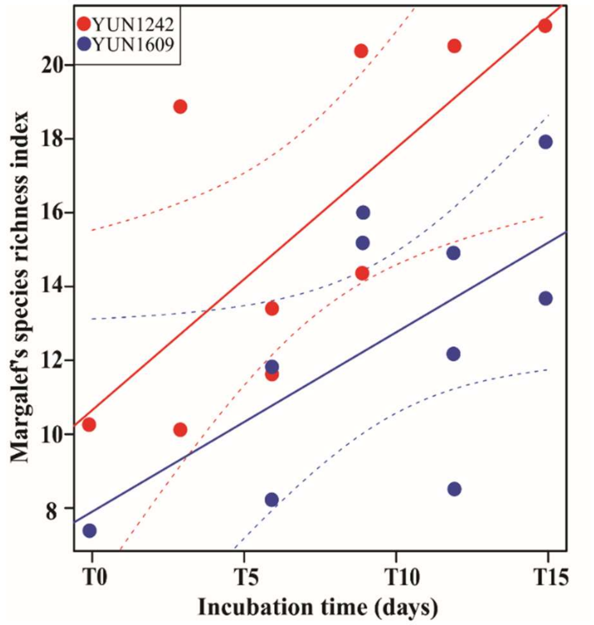

Figure S6. Richness (Margalef) index of the samples from the wetting experiments with YUN1242 and YUN1609 soil pit samples vs incubation time. Regression lines and associated 95% confidence bands are depicted.

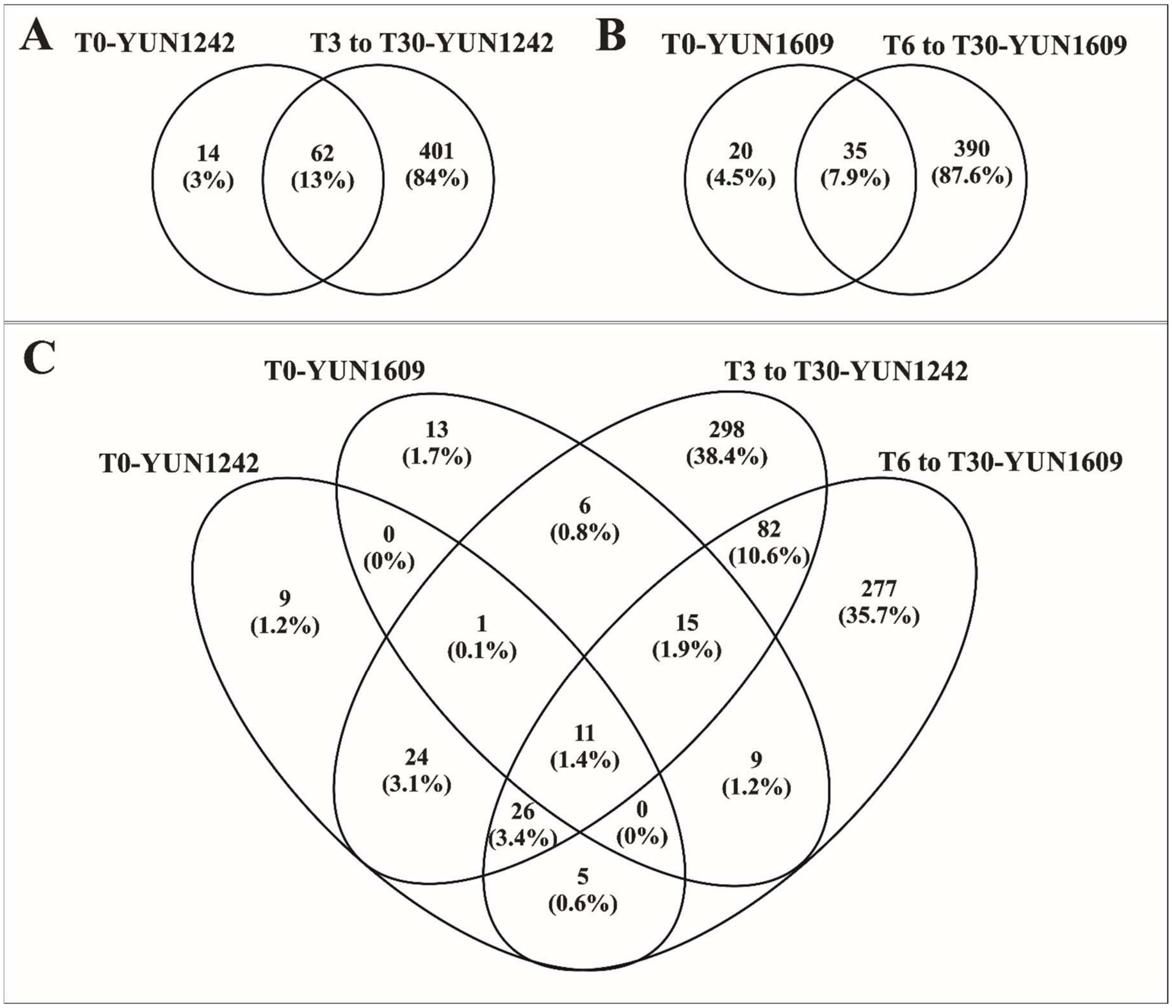

Figure S7. Diagram of the Amplicon Sequence Variants (ASVs) between T0 and the rest of incubation time for (A) YUN1242 samples only, (B) YUN1609 samples only, (C) both sites analyzed together. Additional information in Table S5.

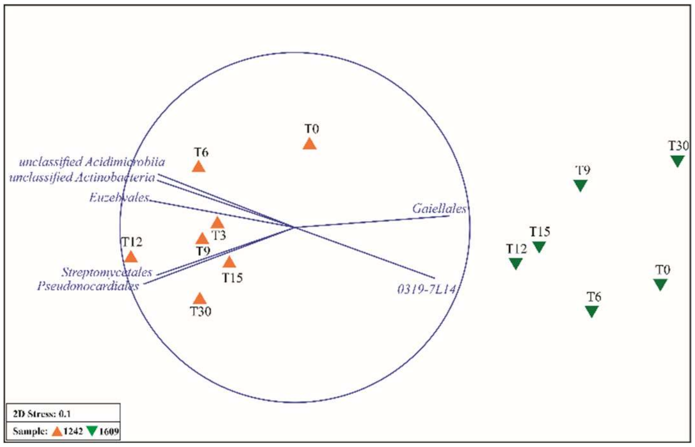

Figure S8. Non-metric multidimensional scaling for *Actinobacterial* orders with vectors overlay of the order that showed correlation (Spearman > 0.75) with the ordination axes.

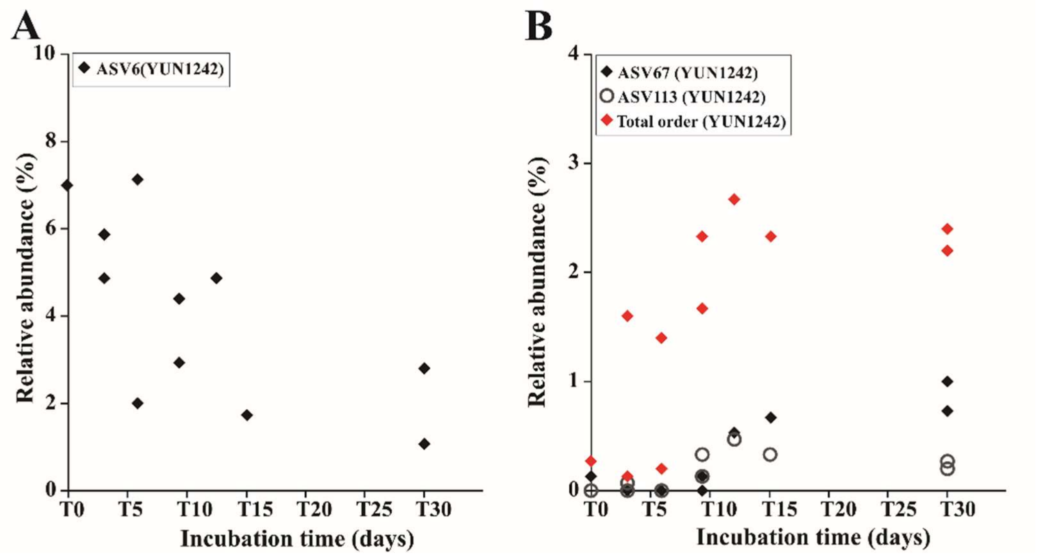

Figure S9. Relative abundance dynamics, during the wetting experiment with YUN1242, of taxa from A) *Parviterribacter* genus; and B) ASVs from *Frankiales* order. Note that each point represents the relative abundance in a single replicated flask.

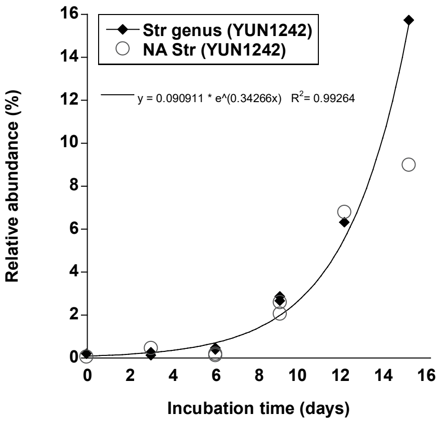

Figure S10. Modelled relative abundance dynamics of *Streptomyces* genus from 0-15 incubation days. Note that each point represents the relative abundance in a single replicated flask.

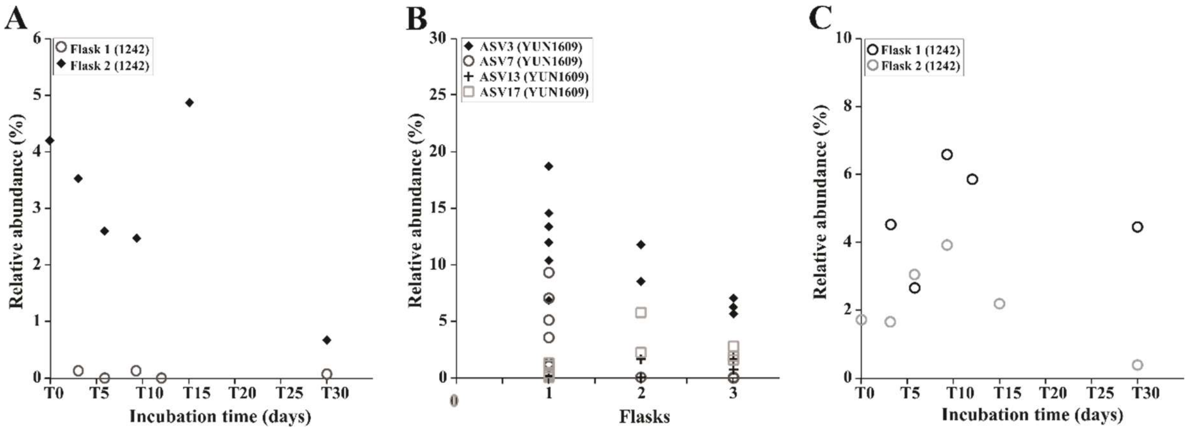

Figure S11. A) *Rubrobacter* ASV37 during the experiment with YUN1242 sample in both replicated flasks. B) ASVs from *Gaiellales* order during the experiment with YUN1609 sample in three replicated flasks. C) ASVs from *Gemmatimonadota* during the experiment with YUN1242 sample in both replicated flasks.

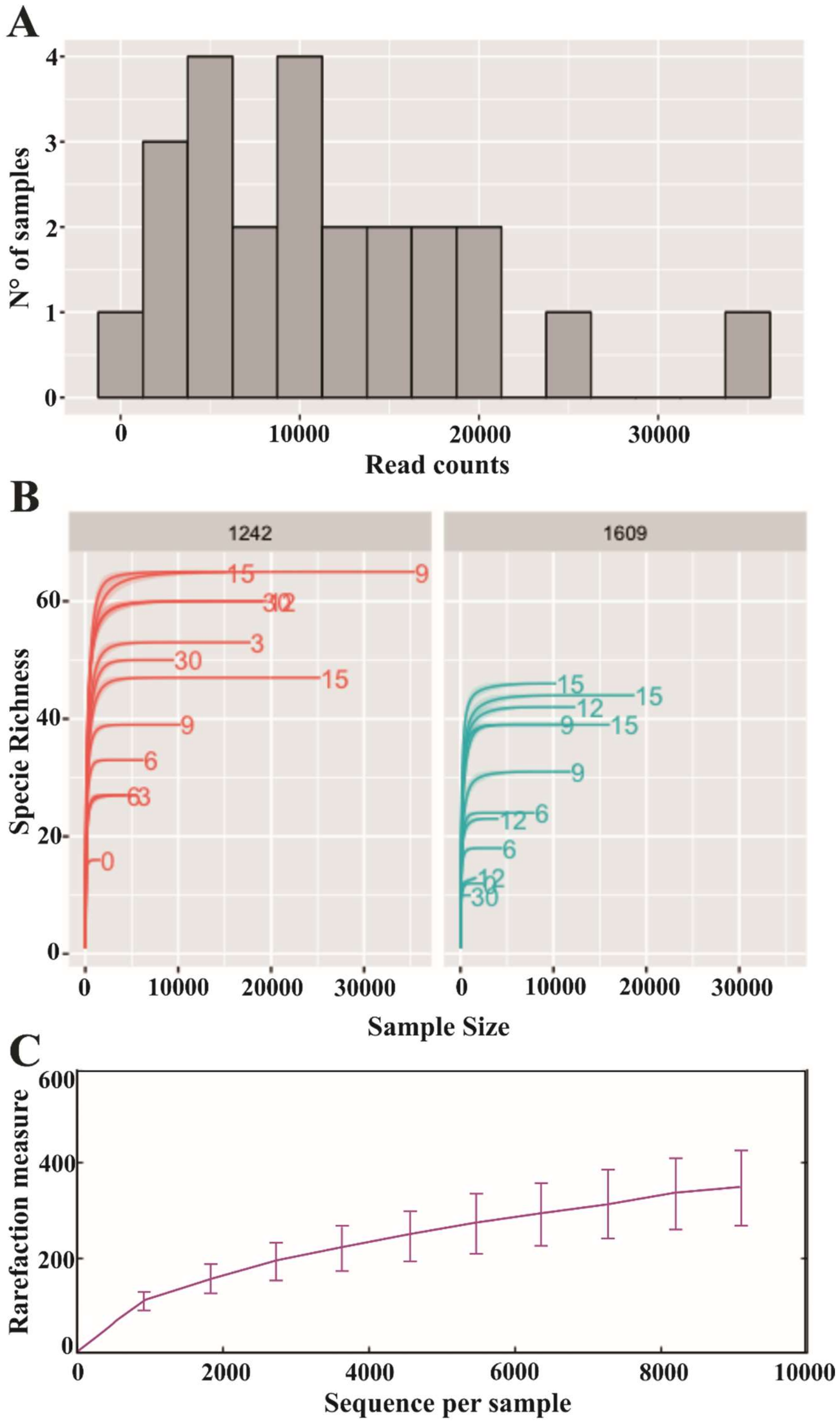

Figure S12. Histogram of distribution of sample sequencing depth vs reads counts (A), rarefaction curves of species richness by number of sequences per target samples (B), and rarefaction measure (observed ASVs) vs sequence per sample (C).

Table S1. Incubation time pairwise multiple comparisons using FDR correction for bacterial abundance data per sampling site determined by qPCR.

		YUN1242 Bacteria				YUN1609 Bacteria			
Group 1	Group 2	Statistics	Df	P	Adjusted P (FDR)	Statistic	Df	P	Adjusted P (FDR)
T0	T3	-1.318	3	0.279	0.438	-4.029	3	0.028	0.058
T0	T6	-1.229	3	0.307	0.438	-5.939	3	0.010	0.040
T0	T9	-2.360	3	0.099	0.298	-3.062	3	0.055	0.082
T0	T12	-0.395	3	0.719	0.755	-1.328	3	0.276	0.362
T0	T15	-2.442	3	0.092	0.298	-4.665	3	0.019	0.049
T0	T30	-10.378	3	0.002	0.040	-5.265	3	0.013	0.041
T3	T6	1.313	3	0.28	0.438	0.779	3	0.493	0.559
T3	T9	-2.048	3	0.133	0.310	-0.753	3	0.506	0.559
T3	T12	0.578	3	0.604	0.668	8.082	3	0.004	0.021
T3	T15	-1.277	3	0.291	0.438	-3.312	3	0.045	0.078
T3	T30	-2.939	3	0.061	0.278	-0.443	3	0.688	0.720
T6	T9	-3.151	3	0.051	0.278	-0.875	3	0.446	0.551
T6	T12	-0.107	3	0.922	0.922	11.641	3	0.001	0.014
T6	T15	-2.070	3	0.130	0.310	-3.367	3	0.044	0.078
T6	T30	-6.858	3	0.006	0.067	-2.499	3	0.088	0.123
T9	T12	0.954	3	0.410	0.490	5.219	3	0.014	0.041
T9	T15	-0.932	3	0.420	0.490	-4.120	3	0.026	0.058
T9	T30	-2.832	3	0.066	0.278	0.393	3	0.720	0.720
T12	T15	-1.148	3	0.334	0.438	-8.631	3	0.003	0.021
T12	T30	-1.844	3	0.162	0.340	-21.126	3	<0.001	0.005
T15	T30	-1.155	3	0.332	0.438	3.225	3	0.048	0.078

Group 1 & Group 2: Incubation time group data to be compared; Statistic: Test statistic used to compute the p-value; Df: Degrees of freedom; P: p-value; Adjusted P (FDR): Adjusted p-value using False Discovery Rate (FDR) correction.

Table S2. Incubation time pairwise multiple comparisons using FDR correction for archaeal abundance data per sampling site determined by qPCR.

		YUN1242 Archaea				YUN1609 Archaea			
Group 1	Group 2	Statistic	Df	P	Adjusted P (FDR)	Statistic	Df	P	Adjusted P (FDR)
T0	T3	-1.172	3	0.326	0.685	-1.950	3	0.146	0.170
T0	T6	-1.290	3	0.287	0.670	-3.052	3	0.055	0.089
T0	T9	-1.359	3	0.267	0.670	-2.484	3	0.089	0.117
T0	T12	-0.291	3	0.79	0.934	0.592	3	0.596	0.626
T0	T15	-0.399	3	0.717	0.934	-3.185	3	0.050	0.087
T0	T30	5.426	3	0.012	0.082	6.473	3	0.007	0.017
T3	T6	0.904	3	0.433	0.827	0.037	3	0.973	0.973
T3	T9	-0.004	3	0.997	0.997	-2.310	3	0.104	0.128
T3	T12	0.680	3	0.545	0.904	7.471	3	0.005	0.013
T3	T15	0.556	3	0.617	0.904	-3.425	3	0.042	0.080
T3	T30	4.980	3	0.016	0.082	16.118	3	<0.001	0.004
T6	T9	-1.549	3	0.219	0.657	-1.640	3	0.200	0.221
T6	T12	0.227	3	0.835	0.934	9.005	3	0.003	0.012
T6	T15	0.213	3	0.845	0.934	-2.603	3	0.080	0.112
T6	T30	10.239	3	0.002	0.042	32.680	3	<0.001	0.001
T9	T12	0.605	3	0.588	0.904	7.441	3	0.005	0.013
T9	T15	0.508	3	0.646	0.904	-2.626	3	0.079	0.112
T9	T30	6.432	3	0.008	0.080	12.822	3	0.001	0.005
T12	T15	-0.015	3	0.989	0.997	-4.591	3	0.019	0.041
T12	T30	1.697	3	0.188	0.657	17.892	3	<0.001	0.004
T15	T30	2.003	3	0.139	0.584	7.633	3	0.005	0.013

Group 1 & Group 2: Incubation time group data to be compared; Statistic: Test statistic used to compute the p-value; Df: Degrees of freedom; P: p-value; Adjusted P (FDR): Adjusted p-value using False Discovery Rate (FDR) correction.

Table S3. Total number of reads per sample.

Samples	Reads input	Reads output (*)	Samples	Reads input	Reads output (*)
T0-1242-1	2685	1452	T0-1609-1	4617	3233
T0-1242-2	3903	2462	T0-1609-2	1767	1049
			T0-1609-3	690	152
T3-1242-1	23633	19508	T3-1609-1	1593	1083
T3-1242-2	8315	6487	T3-1609-2	2335	1256
			T3-1609-3	493	132
T6-1242-1	7134	5446	T6-1609-1	13071	9885
T6-1242-2	9154	7333	T6-1609-2	7026	5191
			T6-1609-3	949	342
T9-1242-1	45284	37811	T9-1609-1	17603	14168
T9-1242-2	14404	11837	T9-1609-2	1094	480
			T9-1609-3	16316	13201
T12-1242-1	26213	21413	T12-1609-1	3919	2686
T12-1242-2	2105	1075	T12-1609-2	6474	4950
			T12-1609-3	17979	14417
T15-1242-2	23511	19155	T15-1609-1	22299	18376
			T15-1609-3	25780	21505
T30-1242-1	30412	24268	T30-1609-1	2812	1894
T30-1242-2	26272	21076	T30-1609-2	410	141
			T30-1609-3	2123	1200

Experimental control	Reads input	Reads output (*)	PCR control	Reads input	Reads output (*)
T0 blank	689	256	Ctrl1	819	64
T3 blank	1467	24	Ctrl2	603	85
T6 blank	594	465	Ctrl3	1002	142
T9 blank	605	973	Ctrl4	1227	201
T12 blank	555	157	Ctrl5	572	34
T15 blank	1355	116	Ctrl6	738	81
T30 blank	1599	167			

**Samples with < 1500 reads output were eliminated. Read output is the number of reads after the quality filtering.*

Table S4. Summary of average diversity indices of both soil pits (A) and by replicates and time for the studied samples (B).

A)

Soil pit ID	Total species (S)	Total individuals (N)	Species richness index (Margalef)	Diversity index (Shannon, H')
YUN1242	115	1500	15.56	2.93
YUN1609	89	1500	12.03	2.69

B)

Soil pit ID	Total Species (S)	Total individuals (N)	Species richness index (Margalef)	Evenness index (Pielou's, J')	Diversity index (Shannon, H')	Dominance index (Simpson, 1-λ')
**T0-YUN1242-2**	76	1500	10.26	0.67	2.9	0.86
**T3-YUN1242-1**	139	1500	18.87	0.59	2.92	0.81
**T3-YUN1242-2**	75	1500	10.12	0.58	2.49	0.75
**T6-YUN1242-1**	99	1500	13.4	0.6	2.76	0.8
**T6-YUN1242-2**	86	1500	11.62	0.61	2.7	0.78
**T9-YUN1242-1**	150	1500	20.37	0.62	3.1	0.84
**T9-YUN1242-2**	106	1500	14.36	0.6	2.8	0.78
**T12-YUN1242-1**	151	1500	20.51	0.66	3.33	0.89
**T15-YUN1242-1**	128	1500	17.37	0.57	2.79	0.83
**T30-YUN1242-1**	142	1500	19.28	0.71	3.51	0.93
**T30-YUN1242-2**	111	1500	15.04	0.62	2.91	0.89
**T0-YUN1609-1**	55	1500	7.38	0.61	2.43	0.81
**T6-YUN1609-1**	87	1500	11.76	0.59	2.66	0.84
**T6-YUN1609-2**	61	1500	8.2	0.56	2.29	0.78
**T9-YUN1609-1**	112	1500	15.18	0.6	2.84	0.83
**T9-YUN1609-3**	118	1500	15.99	0.57	2.74	0.8
**T12-YUN1609-1**	63	1500	8.48	0.66	2.74	0.87
**T12-YUN1609-2**	90	1500	12.17	0.6	2.69	0.82
**T12-YUN1609-3**	110	1500	14.91	0.62	2.9	0.84
**T15-YUN1609-1**	101	1500	13.67	0.55	2.55	0.8
**T15-YUN1609-3**	132	1500	17.91	0.61	2.96	0.86
**T30-YUN1609-1**	50	1500	6.7	0.7	2.74	0.89

Box 1. Detection and removal of contaminants in sequencing data

Methodology: The accuracy of microbial community surveys based on universal marker genes suffers from the presence of contaminants—DNA sequences not truly present in the sample that can come from various sources, including reagents (Kennedy et al., 2023). In this study, nested-PCR (NPCR) of blank and control samples was used to increase the detection sensitivity of any possible contamination. Blank and control samples were amplified using universal bacterial (341F: 5'-CCTACGGGNGGCWGCAG-3', 1070R: 5'-AGCTGACGACAGCCAT-3') and archaeal primers (340F:5'-CCCTAHGGGGYGCASCA-3', 915R: 5'-GWGCYCCCCCGYCAATTC-3'). NPCR was conducted in 50 µL reaction volume containing 10-50 ng/µL of genomic DNA, 0.3 µM of each primer, 0.2 mM of dNTPs, 2 mM of MgCl2, 5% of DMSO, 1 X of colorless Go Taq flexi buffer (Promega M3001, Madison, WI), 1,5 U/µL de Hot Start Taq DNA polymerase (Promega M3001, Madison, WI), and free-nuclease water. The bacterial amplification program consisted of initial denaturation of 95°C for 10 min, 30 cycles of 95 ºC for 30 s of denaturation, 55 ºC for 30 s of annealing, and 72 ºC for 20 s of extension, and final extension of 72°C for 6 min. The archaeal amplification program consisted of initial denaturation of 95°C for 5 min, 20 cycles of 95 ºC for 30 s of denaturation, 61 ºC for 30 s of annealing, and 72 ºC for 20 s of extension, and final extension of 72°C for 10 mi. Those amplicons were sequencing exactly as described in "Diversity through 16S rRNA sequencing" section. The raw reads were included in the same procedure of other samples following the "Taxonomic and Phylogenetic Analysis".

Results: Although, direct PCR amplicon sequencing (control tubes) and DNA extraction (blanks tubes) reagents did not generate sequences that passed the quality score, the nested PCR detected 14 ASVs that represented less than 1% of average abundance (from 2 to 113 ASVs counts) in target samples (Table S1). Some of those ASVs were previously reported as common contaminants in other studies like *Bradyrhizobium, Afipia* (Mishra et al., 2021), *Methylobacterium-Methylorubrum, Haemophilus*, *Lactococcus*, (Longo, O'Neill & O'Neill, 2011), *Renibacterium* (Brown et al., 1994), *Streptococcus* (Salter et al., 2018), and *Cutibacterium* (Kennedy et al., 2021). However, other ASVs were detected as contaminants in this study like *Neisseria*, *Staphylococcus*, *Rhizorhapis*, unclassified *Comamonadaceae*, *Porphyrobacter*, *Solirubrobacter*, and *Acinetobacter* genus. Those ASVs were removed from the sequences of target samples before rarefaction.

Sequence read counts and relative abundance of contaminant ASVs in the target samples, DNA extraction (blanks) and sequencing reagents (controls).

PCR type	Samples \ ASV assigned genus and number	*Acinetobacter*	*Bradyrhizobium*	*Rhizorhapis*	*unclassified Comamonadaceae*	*Methylobacterium-Methylorubrum*	*Porphyrobacter*	*Afipia*	*Cutibacterium*	*Streptococcus*	*Solirubrobacter*	*Neisseria*	*Haemophilus*	*Staphylococcus*	*Lactococcus*	Contaminant ASVs	Sum of ASV by sample	Relative abundance (%)
		ASV9	ASV20	ASV23	ASV 19	ASV 26	ASV 18	ASV 38	ASV 29	ASV 57	ASV 58	ASV 69	ASV 72	ASV 167	ASV 178			
Direct PCR	T0_1242	1	1	0	0	1	3	0	0	0	1	1	1	0	0	9	1,173	0.8
	T0_1242	0	0	0	0	0	0	0	0	0	0	51	0	0	0	51	1,983	2.6
	T3_1242	2	2	0	10	0	1	9	1	12	4	0	1	0	0	42	18,746	0.2
	T3_1242	1	0	0	0	0	0	0	0	0	1	0	0	0	0	2	5,979	0.0
	T6_1242	0	0	0	5	0	0	0	2	0	0	4	0	47	0	58	4,742	1.2
	T6_1242	1	0	0	1	1	1	1	1	0	1	0	0	0	0	7	6,559	0.1
	T9_1242	3	1	1	20	0	2	1	1	0	8	1	0	0	0	38	36,476	0.1
	T9_1242	1	0	0	2	1	2	0	2	0	0	0	0	0	0	8	11,190	0.1
	T12_1242	4	1	0	1	1	2	0	1	1	1	0	0	1	0	13	20,309	0.1
	T12_1242	0	9	5	0	0	0	0	2	0	0	0	0	0	0	16	1,111	1.4
	T15_1242	5	0	0	4	0	5	0	4	26	4	0	8	44	0	100	26,570	0.4
	T15_1242	1	0	0	5	0	0	0	0	0	2	0	0	0	0	8	18,244	0.0
	T30_1242	5	0	0	1	0	1	0	1	0	10	0	1	3	0	22	22,791	0.1
	T30_1242	4	0	0	4	0	2	0	5	1	2	0	1	0	0	19	19,894	0.1
	T0_1609	0	0	0	0	0	0	0	1	0	0	0	0	33	2	36	2,713	1.3
	T3_1609	1	1	3	0	0	0	0	5	0	5	1	0	24	0	40	1,196	3.3
	T6_1609	1	0	1	1	1	1	0	0	0	0	0	0	57	0	62	8,761	0.7
	T6_1609	3	0	0	6	0	0	0	0	0	0	0	0	0	0	9	4,943	0.2
	T9_1609	1	0	1	1	0	0	0	0	10	0	1	0	44	0	58	12,770	0.5
	T9_1609	10	1	15	7	0	0	1	2	8	0	3	5	0	0	52	12,421	0.4
	T12_1609	1	0	0	0	1	0	0	0	0	0	0	0	0	0	2	1,905	0.1
	T12_1609	2	0	0	0	0	0	0	0	0	0	0	0	0	0	2	4,524	0.0
	T12_1609	3	2	0	1	0	2	0	0	1	0	0	0	0	0	9	13,027	0.1
	T15_1609	8	4	2	0	0	0	0	1	0	1	11	5	0	0	32	17,415	0.2
	T15_1609	1	0	1	19	0	0	0	1	0	6	0	0	0	0	28	10,999	0.3
	T15_1609	2	0	0	2	0	0	5	2	0	4	2	0	1	0	18	20,029	0.1
	T30_1609	0	0	0	0	0	1	0	0	59	0	0	0	0	0	60	1,251	4.8
	T30_1609	45	0	0	0	0	0	0	0	31	0	13	22	2	0	113	1,170	9.7

Sequence read counts and relative abundance of contaminant ASVs in the target samples, DNA extraction (blanks) and sequencing reagents (controls). (***continuation***).

PCR type	Samples \ ASV assigned genus and number	Acinetobacter	Bradyrhizobium	Rhizorhapis	unclassified Comamonadaceae	Methylobacterium-Methylorubrum	Porphyrobacter	Afipia	Cutibacterium	Streptococcus	Solirubrobacter	Neisseria	Haemophilus	Staphylococcus	Lactococcus	Contaminant ASVs	Sum of ASV by sample	Relative abundance (%)
		ASV9	ASV20	ASV23	ASV 19	ASV 26	ASV 18	ASV 38	ASV 29	ASV 57	ASV 58	ASV 69	ASV 72	ASV 167	ASV 178			
NPCR	T6 blank	25,665	4	2	6	3	9	1	1	21,263	0	13,407	12,610	0	2	72,973	73,773	98.9
	T9 blank	4	1	4	3	47,485	4	0	13,198	3	0	0	0	0	0	60,702	61,040	99.4
	T15 blank	3	57,407	6	3	6	5	3	0	2	1	0	0	0	0	57,436	57,664	99.6
	T30 blank	0	0	0	9	0	0	0	28	56	0	0	0	581	350	1,024	1,058	96.8
	Control 2	1	3	0	1	2	3	36,163	0	1	1	0	0	0	0	36,175	36,333	99.6
	Control 3	56,584	3	21,736	21,593	0	7	5	3	3	19,791	0	23	0	0	119,748	120,415	99.4
	Control 4	8	0	3	27,313	0	1	0	7,530	0	3	0	0	0	0	34,858	35,350	98.6
	Control 5	4	6	3	10	0	45,174	0	8,638	0	0	1	0	0	0	53,836	54,143	99.4
	Control 6	19	0	30,533	1	1	3	0	0	1	0	0	1	1	4	30,564	30,693	99.6
	Sum of ASV reads in target samples	106	22	29	90	6	23	17	32	149	50	88	44	256	2			
	Sum of ASV reads	82,394	57,446	52,316	49,029	47,503	45,229	36,189	29,430	21,478	19,846	13,496	12,678	838	358			
	Mean abundance in target samples (%)	0.12865	0.03830	0.05543	0.18356	0.01263	0.05085	0.04698	0.10873	0.69373	0.25194	0.65205	0.34706	30.54893	82.23615			

Table S5. Taxa represented in the ASVs diagram (Fig. S5).

ASV counts	ASV Abundance (%)	Interpretation<br>ASVs are…	Interpretation	Genus associated to ASVs
9	1.2	present only in "T0-1242"	ASVs not found after wetting	Akkermansia (asv807), U. Rhodocyclaceae Family (asv1116), Rheinheimera (asv1215), Gaiella (asv1426), C39 (asv891), U. Euzebyaceae Family (asv204, asv964), U. Acidimicrobiia Class (asv930), U. Planococcaceae Family (asv1347)
13	1.7	present only "T0-1609"		Leucobacter (asv926), Extensimonas (asv1111), U. Comamonadaceae Family (asv1035, asv1571, asv1558), Hyphomicrobium (asv1192, asv1149, asv1315), U. MB-A2-108 Class (asv1244), Hephaestia (asv1410), Lactococcus (asv233), U. Euzebyaceae Family (asv1457), Rubrobacter (asv1475)
24	3.1	present in "T0-1242" and "T3 to T30- 1242"	ASVs detected in both initial and incubation stages. It means that ASVs could persist or even replicate during the wetting experiment.	U. Chloroflexi Phylum (asv264), U. Longimicrobiaceae Family (asv424, asv107, asv357), U. 0319-7L14 Order (asv220, asv87), U. AKYG1722 Family (asv109), Rubrobacter (asv186), U. 67-14 Family (asv406), U. Gaiellales Order (asv215, asv475, asv274), U. Acidimicrobiia Class (asv214), U. Nitriliruptoraceae Family (asv108, asv283, asv358), U. Frankiales Order (asv79), Conexibacter (asv81), U. Actinobacteria Class (asv111, asv346), U. Chloroflexi Phylum (asv403), U. Vicinamibacterales Order (asv687), Parviterribacter (asv331), Kallotenue (asv847)
9	1.2	present in "T0-1609" and "T6 to T30- 1609"		U. 67-14 Family (asv97, asv174), U. Armatimonadales Order (asv1352), U. 0319-7L14 Order (asv291, asv384), U. Euzebyaceae Family (asv156), U. Nitriliruptoraceae Family (asv163, asv742), Rubrobacter (asv296)
5	0.6	present in "T0-1242" and "T6 to T30- 1609"		Actinomyces (asv1024), Gemella (asv64), Paracoccus (asv38), U. JG30-KF-CM45 Family (asv153), U. Comamonadaceae Family (asv185)
1	0.1	present in "T0-1242", "T3 to T30- 1242" and "T0-1609"		Kallotenue (asv98)
26	3.4	present in "T0-1242", "T3 to T30- 1242" and "T6 to T30- 1609"		Parviterribacter (asv6), U. Actinobacteria Class (asv8, asv302, asv41, asv100), Rubrobacter (asv39, asv218, asv106), U. Acidimicrobiia Class (asv27), U. Gaiellales Order (asv13, asv43), U. Euzebyaceae Family (asv11, asv30, asv418), Ralstonia (asv44), U. KD4-96 Class (asv104), U. JG30-KF-CM45 Family (asv16), U. 67-14 Family (asv190), U. Nitriliruptoraceae Family (asv195, asv176, asv192), Streptomyces (asv15, asv52), U. Streptomycetaceae Family (asv9), Marmoricola (asv60), U. Frankiales Order (asv784),
15	1.9	present in "T0-1609", "T3 to T30- 1242" and "T6 to T30- 1609"		U. 0319-7L14 Order (asv20), U. 67-14 Family (asv29, asv96, asv538, asv225), Rubrobacter (asv47, asv171, asv19), U. Gaiellales Order (asv7, asv23), U. TK10 Class (asv330), U. JG30-KF-CM45 Family (asv366), U. Nitriliruptoraceae Family (asv76, asv118), Thermobaculum (asv46)
11	1.4	present in "T0-1242", "T0-1609", "T3 to T30- 1242" and "T6 to T30- 1609"		U. Nitriliruptoraceae Family (asv2, asv1, asv141), Rubrobacter (asv4, asv5, asv94), U. Gaiellales Order (asv3), U. Longimicrobiaceae Family (asv14), U. Nocardioidaceae Family (asv24), Thermobaculum (asv10), U. Frankiales Order (asv37)
6	0.8	present in "T0-1609" and "T3 to T30- 1242"		Rheinheimera (asv1117), Streptomyces (asv56), U. Nitriliruptoraceae Family (asv142), U. Actinobacteria Class (asv50), U. 67-14 Family (asv116, asv705)
298	38.4	present only in "T3 to T30- 1242"	ASVs detected only by a suitable environment	Streptomyces (asv28, asv132, asv1418, asv277), U. Longimicrobiaceae Family (asv63, asv55, asv216, asv235, asv184, asv1095, asv881, asv454, asv730, asv544, asv1271, asv1480), U. Gitt-GS-136 Class (asv67, asv294, asv703, asv493, asv1230), U. Euzebyaceae Family (asv295, asv324, asv201, asv372, asv319, asv129, asv259, asv485, asv158, asv269, asv434, asv781, asv146, asv299, asv226, asv451, asv592, asv722, asv801, asv876, asv1163, asv1395, asv1519, asv1605), Rubrobacter (asv122, asv182, asv187, asv310, asv611, asv425, asv549, asv377, asv966, asv212, asv698, asv819, asv896, asv913, asv1081), U. Acidimicrobiia Class (asv1394, asv198, asv170, asv566, asv570, asv243, asv322, asv409, asv593, asv221, asv1394), Quadrisphaera (asv181), U. 0319-7L14 Order (asv160, asv124, asv805, asv1218, asv1393, asv1562, asv338, asv1250), Nitrospira (asv1037, asv982), U. 67-14 Family (asv69, asv609, asv161, asv382, asv1039, asv684, asv873, asv1050, asv1143, asv1382, asv1417, asv1566, asv1574), U. Streptomycetaceae Family (asv123, asv367, asv1603), U. Frankiales Order (asv562, asv159, asv208, asv470, asv435, asv543, asv631, asv1118, asv731, asv1282), Lechevalieria (asv604, asv179), U. WD2101 soil group Family (asv253), Crossiella (asv590), Zoogloea (asv1038, asv1142), Cryptosporangium (asv207, asv523, asv773), Herminiimonas (asv1032), hgcI clade (asv1194), U. Sporichthyaceae Family (asv200, asv557), U. Comamonadaceae Family (asv1304, asv1198, asv1348, asv1577, asv1223, asv1604, asv1346, asv1553), Conexibacter (asv139, asv247, asv605, asv1196, asv636, asv284, asv738), Rheinheimera (asv1252), U. Azospirillaceae Family (asv1286), Truepera (asv135, asv649), U. IMCC26256 Order (asv230), Paracoccus (asv1031), Cupriavidus (asv1069), Klenkia (asv328), Gaiella (asv396, asv715, asv894, asv1004, asv364), U. Bacteria kingdom (asv480), U. Subgroup 7 Order (asv315), Aridibacter (asv478), Prevotella (asv1211), U. AKYG1722 Family (asv188), U. JG30-KF-CM45 Family (asv143, asv267, asv271, asv336, asv189, asv614, asv1007, asv1046), Nocardioides (asv276, asv419), Blastococcus (asv506, asv860), U. Gaiellales Order (asv1318, asv712, asv393, asv327, asv371, asv869), U. Ezakiella Family (asv1433), U. Nitriliruptoraceae Family (asv75, asv292, asv479, asv654, asv816, asv743, asv510, asv826, asv846, asv1202, asv1262, asv1506, asv1596), Ellin6055 (asv1222, asv1399), Devosia (asv1390), Pseudonocardia (asv313, asv473, asv849, asv1174), U. Thermomicrobiaceae Family (asv370), U. Cyclobacteriaceae Family (asv507, asv89), Abditibacterium (asv588), Sphingomonas (asv827, asv36), U. Enterobacteriaceae Family (asv1137), Hydrogenophaga (asv1200, asv1579), Candidatus Nitrocosmicus (asv213), U. Pseudonocardiaceae Family (asv239, asv1297), U. TK10 Class (asv939, asv99, asv197, asv1458, asv1477, asv1501), Acidithiobacillus (asv1361), Corynebacterium (asv1378), U. SHA-26 Class (asv642), U. Bacillaceae Family (asv670), Actinocatenispora (asv1064), U. Gammaproteobacteria Class (asv1220), [Ruminococcus] gnavus group (asv1313), Aquabacterium (asv1356, asv1552), Nakamurella (asv343, asv447), Amycolatopsis (asv666), U. Isosphaeraceae Family (asv1212, asv355, asv1121), Pseudoxanthomonas (asv1478), Kribbella (asv280), U. AKIW781 Family (asv320, asv893, asv577, asv878), U. Actinobacteria Class (asv392, asv270, asv603, asv757, asv934), Candidatus Nitrososphaera (asv514, asv255, asv363), Nitrolancea (asv563), YC-ZSS-LKJ147 (asv820, asv468), U. Rhodocyclaceae Family (asv1232), U. Muribaculaceae Family (asv1377), Thermobaculum (asv178, asv852), U. Rhizobiales Order (asv205, asv1524), Abditibacterium (asv260), Undibacterium (asv547), U. Micromonosporaceae Family (asv607, asv362, asv645, asv917), U. Gemmataceae Family (asv658), U. Chloroflexi Phylum (asv997, asv806), Actinophytocola (asv1327), Bifidobacterium (asv1408), Pseudorhodoferax (asv1443), U. Pedosphaeraceae Family (asv1447, asv1508), Glycomyces (asv1541), Parviterribacter (asv282, asv391, asv726, asv369), Psychroglaciecola (asv520), U. Myxococcaceae Family (asv681, asv621), Actinotalea (asv872), Peredibacter (asv995), U. JG30-KF-CM66 Class (asv1126, asv1251, asv627), Roseisolibacter (asv1217), Tetragenococcus (asv1528), Shinella (asv1551), U. Solirubrobacteraceae Family (asv209, asv943), Tundrisphaera (asv236), Ramlibacter (asv307), U. Thermomicrobiales Order (asv415), Candidatus Udaeobacter (asv423), Geodermatophilus (asv533), Actinomyces (asv691), Gemmatimonas (asv793), Allostreptomyces (asv814), Pelomonas (asv825), Kibdelosporangium (asv950), Segetibacter (asv969), Stenotrophomonas (asv1074), Enhydrobacter (asv1103), Burkholderia-Caballeronia-Paraburkholderia (asv1343), U. Elev-16S-1166 Order (asv1462), Parabacteroides (asv1464), Ferribacterium (asv1473), U.
277	35.7	present only in "T6 to T30- 1609"		U. LiUU-11-161 Family (asv892), Dongia (asv850), Zoogloea (asv864), U. Rhizobiales Incertae Sedis Family (asv867), Ferrovibrio (asv1141), U. Microscillaceae Family (asv351, asv210, asv26), U. Saprospiraceae Family (asv1092), U. Rhizobiales Order (asv1005), Rheinheimera (asv989, asv1139, asv1580), Pedobacter (asv1077), Shewanella (asv1006), U. 0319-7L14 Order (asv84, asv266, asv692, asv164, asv113, asv756, asv273, asv439, asv1065, asv1178, asv1581), U. KD4-96 Class (asv242, asv709, asv542, asv460, asv456), U. Comamonadaceae Family (asv1101, asv1530, asv1329), Ellin6067 (asv1088), Rhodoferax (asv1254), U. Bacillaceae Family (asv1033), Rubrobacter (asv248, asv217, asv133, asv42, asv193, asv219, asv289, asv559, asv996, asv125, asv380, asv381, asv540, asv618, asv623, asv729, asv824, asv937, asv1490, asv1489, asv1470, asv1455, asv1360, asv1342, asv1336, asv1324, asv1067asv131), U. Saprospiraceae Family (asv732), Thermobaculum (asv77, asv340, asv194), U. TK10 Class (asv689, asv486, asv474, asv1134, asv1185, asv1526), U. Euzebyaceae Family (asv101, asv73, asv823, asv448, asv231, asv1569, asv1522, asv1484, asv1320), U. 67-14 Family (asv147, asv70, asv643, asv1209, asv751, asv575, asv386, asv554, asv1365, asv127, asv462, asv541, asv1096, asv1556, asv1517, asv1513, asv1516), U. Gaiellales Order (asv102, asv167, asv240, asv513, asv33, asv261, asv250, asv311, asv180, asv443, asv196, asv173, asv287, asv379, asv1337, asv508, asv334, asv337, asv741, asv786, asv904, asv938, asv1307, asv608), Bryocella (asv1289), SH-PL14 (asv929), U. Proteobacteria Phylum (asv1416), Aeromonas (asv1124), U. Gemmataceae Family (asv978), Lactobacillus (asv1308), Corynebacterium (asv1432), Mycobacterium (asv831), U. Armatimonadota Phylum (asv1057), U. Acidimicrobiia Class (asv254, asv416, asv376, asv303, asv620, asv1132, asv582, asv457, asv661, asv672, asv1043), U. Actinobacteriota Phylum (asv453, asv1611), U. Actinobacteria Class (asv512, asv165, asv234), Candidatus Portiera (asv924), Parviterribacter (asv82, asv1527, asv1021), Odoribacter (asv1193), Kinneretia (asv1272), U. IMCC26256 Order (asv172, asv427, asv718, asv545), U. Muribaculaceae Family (asv1491, asv1560, asv1388, asv1189), Wohlfahrtiimonas (asv1268), U. Sporichthyaceae Family (asv1452), U. Gemmatimonadaceae Family (asv1472), U. S0134 terrestrial group Class (asv138), Cloacibacterium (asv940), Bacteroides (asv348, asv1166), Undibacterium (asv1335, asv1225), Nitrospira (asv1261, asv130), Gaiella (asv432, asv1047, asv859, asv1290, asv1383, asv610), U. MBNT15 Phylum (asv657), Salinibacter (asv1213), U. Oscillospiraceae Family (asv1326), U. Thermomicrobiales Order (asv85, asv398), U. JG30-KF-CM45 Family (asv509, asv304, asv1028, asv647, asv349), U. AKIW781 Family (asv990), Prevotellaceae UCG-001 (asv1098), U. Moraxellaceae Family (asv1161), Phenylobacterium (asv1179), U. vadinHA49 Class (asv1441), U. Parachlamydiaceae Family (asv1510), Kocuria (asv1557), U. Nitriliruptoraceae Family (asv1294, asv1131, asv241, asv1305, asv496, asv175, asv74, asv134, asv206), U. Solirubrobacterales Order (asv1498, asv561), U. Chloroflexi Phylum (asv928), U. Bacteria kingdom (asv1287), U. Elsterales Order (asv1302), Alloprevotella (asv1404), Lachnospiraceae NK4A136 group (asv1407), Ornithinimicrobium (asv1471), Abditibacterium (asv839, asv1542), Thauera (asv988), Xanthomonas (asv1054), Anoxybacillus (asv1321), Rikenella (asv1389), Candidatus Saccharimonas (asv1431), GCA-900066575 (asv1439), Microvirga (asv1503), Salinimicrobium (asv35), Porphyromonas (asv49), U. Gitt-GS-136 Class (asv309), Enterococcus (asv1107), Methylocystis (asv1133), Colidextribacter (asv1291), Ottowia (asv1349), U. AKIW781 Family (asv1370), Massilia (asv1385), U. Tissierella Family (asv1413), Sediminibacterium (asv1421), U. Ruminococcaceae Family (asv1430), Thiopseudomonas (asv1459), Mannheimia (asv53), Actinobacillus (asv88), U. 0319-6G20 Order (asv498), U. Peptoniphilus Family (asv568), Burkholderia-Caballeronia-Paraburkholderia (asv585), Nocardioides (asv957, asv1240), Serratia (asv1187), Roseisolibacter (asv1328), U. Frankiales Order (asv1479, asv832, asv1507), Vagococcus (asv1511), Psychroglaciecola (asv1540), Pelosinus (asv1549), Mesorhizobium (asv112), Conexibacter (asv128, asv967), MND1 (asv278), U. Sericytochromatia Class (asv639, asv318), U. Subgroup 2 Order (asv550), Candidatus Nitrososphaera (asv580), Romboutsia (asv606), Gluconobacter (asv688), U. MB-A2-108 Class (asv721), Bosea (asv953), Rothia (asv1100), Aurantisolimonas (asv1113), Luteimonas (asv1152), Puia (asv1298), U. AKIW781 Family (asv1338), Rubellimicrobium (asv1442), U. Candidatus Moranbacteria Order (asv1453), Aerococcus (asv1486), U. P2-11E Class (asv1512), Acidovorax (asv1515), U. Acetobacteraceae Family (asv1529), Sphingobacterium (asv1537), Pseudolabrys (asv1575)
82	10.6	present in "T3 to T30- 1242" and "T6 to T30- 1609"		U. Euzebyaceae Family (asv530, asv32, asv22, asv48), Pseudonocardia (asv223, asv51, asv157), Conexibacter (asv80), U. Gaiellales Order (asv59, asv65, asv17, asv412, asv497, asv191), U. 0319-7L14 Order (asv553, asv45, asv145), Blastococcus (asv162), Thermobaculum (asv78), U. Beijerinckiaceae Family (asv312), U. Isosphaeraceae Family (asv119), Parviterribacter (asv272), Massilia (asv933), U. IMCC26256 Order (asv61, asv203), U. Nocardioidaceae Family (asv144), Rubrobacter (asv57, asv86, asv21, asv202, asv857, asv12, asv92, asv222, asv383), U. KD4-96 Class (asv105), Burkholderia-Caballeronia-Paraburkholderia (asv31), Pseudomonas (asv728), U. 67-14 Family (asv166, asv293, asv433, asv347, asv516, asv136), Candidatus Nitrososphaera (asv18, asv34), U. Microtrichales Order (asv350), U. Sericytochromatia Class (asv62), [Eubacterium] xylanophilum group (asv979), Nitrospira (asv115), Klebsiella (asv572), Acidovorax (asv790), U. JG30-KF-CM45 Family (asv93, asv137, asv121, asv149, asv199), U. TK10 Class (asv246), U. Chloroflexi Phylum (asv227, asv1195, asv745), Conexibacter (asv916), Rhodoferax (asv1208), Micrococcus (asv1449, asv1546), SZB85 (asv25), U. KD4-96 Class (asv40), U. JG30-KF-CM45 Family (), Craurococcus-Caldovatus (asv150), Burkholderia-Caballeronia-Paraburkholderia (asv263, asv634), U. Rhizobiales Order (asv306), U. Frankiales Order (asv329), U. JG30-KF-CM66 Class (asv368), Paucibacter (asv438), U. Acidimicrobiia Class (asv679, asv702), U. Muribaculaceae Family (asv923, asv1048), Kocuria (asv1071), Renibacterium (asv1292), U. Vicinamibacterales Order (asv1314), Bacteroides (asv1322)
776	100	Total		

Table S6. One-way ANOVA analysis of taxon abundance changes during wetting experiments.

**Taxon identification**	**Experiment**	**Factor**	**Groups**	**P-Value**	**F Statistic**	**Figure**
Streptomyces Genus	YUN1242	Inc. time	1. 0-12 days, 2. 15-30 days	0.006	12.99	Fig. 5A
Nitriliruptoraceae ASV2	YUN1242	Inc. time	1. 0-9 days, 2. 12-30 days	0.0004	29.33	Fig. 5B
Nocardiodales	YUN1242	Inc. time	1. 0-9 days, 2. 15-30 days	0.015	11.48	Fig. 5C
Nocardiodales	YUN1609	Inc. time	1. 0-12 days, 2. 15-30 days	0.014	9.12	Fig. 5C
Parviterribacter Genus	YUN1242	Inc. time	1. 0-6 days, 2. 9-30 days	0.05	5.00	Fig. S9A
Frankiales ASV67	YUN1242	Inc. time	1. 0-9 days, 2. 12-30 days	< 0.0001	78.77	Fig. S9B
Rubrobacter ASV37	YUN1242	Flask	1. Flask 1, 2. Flask 2	0.0016	19.80	Fig. S11A
Gaiellales ASV17	YUN1609	Flask	Flask 1, Flask 2, Flask 3	0.012	8.16	Fig. S11B
Gemmatimonadota	YUN1242	Inc. time	1. 0, 3 and 6 days, 2. 9, 12 and 15 days	0.290	1.31	Fig. S11C

***Inc. time= Incubation time.**

Table S7. DNA concentration and bacterial and archaeal abundances by qPCR of samples YUN1242 and YUN1609.

**Sample**	Flask	Replicate	Days	DNA (ng g-¹)	Bacterial abundance (copies g-¹)	Archaeal abundance (copies g -¹)	Log 10 of bacterial abundance	Log 10 of archaeal abundance
1242	1	1	0	4.1	7.66E+05	3.35E+04	5.88	4.53
1242	1	2	0	4.1	3.18E+06	4.14E+04	6.50	4.62
1242	1	1	3	19.2	1.48E+07	3.52E+05	7.17	5.55
1242	1	2	3	19.2	1.48E+07	4.59E+05	7.17	5.66
1242	1	1	6	7.6	3.49E+06	1.84E+05	6.54	5.26
1242	1	2	6	7.6	4.80E+06	1.84E+05	6.68	5.26
1242	1	1	9	16.5	1.25E+07	3.31E+05	7.10	5.52
1242	1	2	9	16.5	2.54E+07	3.35E+05	7.40	5.53
1242	1	1	12	6.8	3.02E+07	7.18E+05	7.48	5.86
1242	1	2	12	6.8	4.06E+07	1.01E+06	7.61	6.00
1242	1	1	15	8.1	3.91E+07	8.58E+05	7.59	5.93
1242	1	2	15	8.1	4.99E+07	4.54E+05	7.70	5.66
1242	1	1	30	10.3	4.37E+07	1.08E+04	7.64	4.03
1242	1	2	30	10.3	4.05E+07	1.33E+04	7.61	4.12
1242	2	1	0	3.7	1.50E+06	5.33E+04	6.18	4.73
1242	2	2	0	3.7	1.69E+06	5.40E+04	6.23	4.73
1242	2	1	3	13.1	1.42E+06	2.76E+04	6.15	4.44
1242	2	2	3	13.1	1.16E+06	4.22E+04	6.06	4.63
1242	2	1	6	5.2	1.65E+06	4.35E+04	6.22	4.64
1242	2	2	6	5.2	1.40E+06	4.17E+04	6.15	4.62
1242	2	1	9	18.0	2.37E+06	4.06E+04	6.37	4.61
1242	2	2	9	18.0	2.43E+06	4.19E+04	6.39	4.62
1242	2	1	12	6.6	2.45E+05	7.40E+03	5.39	3.87
1242	2	2	12	6.6	2.12E+05	4.52E+03	5.33	3.66
1242	2	1	15	8.9	1.11E+08	2.78E+03	8.05	3.44
1242	2	2	15	8.9	1.23E+06	2.45E+05	6.09	5.39
1242	2	1	30	8.9	7.32E+07	6.51E+03	7.86	3.81
1242	2	2	30	8.9	7.20E+07	5.57E+03	7.86	3.75
1609	1	1	0	4.4	7.04E+05	1.44E+04	5.85	4.16
1609	1	2	0	4.4	5.85E+05	2.49E+04	5.77	4.40
1609	1	1	3	2.5	1.63E+06	2.72E+04	6.21	4.43
1609	1	2	3	2.5	3.55E+06	1.92E+04	6.55	4.28
1609	1	1	6	3.6	2.55E+06	2.39E+04	6.41	4.38
1609	1	2	6	3.6	2.94E+06	3.73E+04	6.47	4.57
1609	1	1	9	3.1	1.51E+06	2.44E+04	6.18	4.39

1609	1	2	9	3.1	2.33E+06	3.50E+04	6.37	4.54
1609	1	1	12	3.4	5.30E+05	8.30E+03	5.72	3.92
1609	1	2	12	3.4	5.61E+05	8.30E+03	5.75	3.92
1609	1	1	15	6.6	6.96E+06	7.34E+04	6.84	4.87
1609	1	2	15	6.6	6.97E+06	5.85E+04	6.84	4.77
1609	1	1	30	5.0	2.90E+06	1.53E+03	6.46	3.18
1609	1	2	30	5.0	3.06E+06	1.53E+03	6.49	3.18
1609	2	1	0	2.8	1.57E+05	5.76E+03	5.20	3.76
1609	2	2	0	2.8	1.45E+05	1.08E+04	5.16	4.03
1609	2	1	3	8.0	3.66E+06	4.06E+04	6.56	4.61
1609	2	2	3	8.0	2.99E+06	4.62E+04	6.48	4.66
1609	2	1	6	4.7	1.97E+06	3.26E+04	6.29	4.51
1609	2	2	6	4.7	2.12E+06	3.26E+04	6.33	4.51
1609	2	1	9	8.6	6.00E+06	7.41E+04	6.78	4.87
1609	2	2	9	8.6	7.16E+06	7.41E+04	6.85	4.87
1609	2	1	12	5.0	4.94E+05	1.33E+04	5.69	4.12
1609	2	2	12	5.0	7.04E+05	9.26E+03	5.85	3.97
1609	2	1	15	5.7	9.04E+06	1.37E+05	6.96	5.14
1609	2	2	15	5.7	1.15E+07	8.50E+04	7.06	4.93
1609	2	1	30	5.2	3.16E+06	1.61E+03	6.50	3.21
1609	2	2	30	5.2	3.06E+06	1.61E+03	6.49	3.21
